# Devulcanization of Waste Tire Rubber via Microwave and Biological Methods: A Review

**DOI:** 10.3390/polym17030285

**Published:** 2025-01-22

**Authors:** Mostafa Vahdatbin, Pouria Hajikarimi, Ellie H. Fini

**Affiliations:** 1 School of Chemical, Petroleum and Gas Engineering, Iran University of Science and Technology, Tehran 1684613114, Iran; m_vahdatbin@chemeng.iust.ac.ir; 2 Department of Civil & Environmental Engineering, Amirkabir University of Technology (Tehran Polytechnic), Tehran 1591634311, Iran; phajikarimi@aut.ac.ir; 3 School of Sustainable Engineering and the Built Environment, Ira A. Fulton Schools of Engineering, Arizona State University, 660 S. College Avenue, Tempe, AZ 85287-3005, USA

**Keywords:** ground tire rubber, recycling, devulcanization, desulfurization, microwave, biological

## Abstract

This paper presents a thorough literature review on devulcanization methods applied to waste tire rubber: “microwave devulcanization” and “biological desulfurization”. To do so, 80 papers published from the year 1990 to 2024 in journals with subscription and open access status across 12 databases were reviewed. This paper compares the efficacy and reviews the basic concepts, advantages, processes, and variable parameters of these two methods. In microwave devulcanization, microwave energy breaks the sulfur crosslinks between polymer chains. The latter breakage is mainly enabled by the presence of carbon black in the tire, which is an excellent microwave absorbent. In biological desulfurization, bacteria or fungi convert the crosslinks to elemental sulfur substances or sulfate. In general, microwave devulcanization of rubber leads to a lower crosslink density and thus a higher degree of devulcanization. On the one hand, breaking the crosslinks requires a significantly shorter time than biological desulfurization. Crosslink scission occurs throughout the sample in microwave devulcanization but only on the sample surface in biological desulfurization. Microwave devulcanization is not sensitive to rubber additives and does not require detoxification before devulcanization. On the other hand, biological desulfurization requires detoxification before devulcanization since it involves living organisms that may not tolerate certain rubber additives.

## 1. Introduction

Tire rubber belongs to a group of materials referred to as elastomers; they are particular types of polymers with high ultimate elongation, high elastic memory, and low elastic modulus [1]. These polymers are employed in hospitals, the automotive industry, toys, shoes, rubbery clothes, insulating materials, and so on [2].

According to statistical data, the production of tires at the global level increased by 5% in 2011 in comparison with that in 2010, and it reached 14.68 million tons [3]. In 2016, global rubber production reached 26.9 million tons annually. Moreover, the International Rubber Study Group (IRSG) predicted that global rubber consumption would grow at an average annual rate of 2.8% from 2017 to 2025 [4]. A recent report presented by the International Market Analysis Research and Consulting (IMARC) Group claimed that the estimated global size of the tire market was approximately 2.3 billion units in 2022. In addition, according to the prediction of the same report, by 2028, the tire market will amount to 2.7 billion units approximately, with a compound annual growth rate (CAGR) of 2.8% during the 2023–2028 period [5]. According to a recent report by Amin et al., 1.5 billion end-of-life tires (ELTs) are disposed of at the global scale annually [4,6]. This figure would amount to an annual increase of 1.2 billion by the year 2030. Besides the tires stockpiled, 5 billion tires are required to be discarded regularly [7].

It is noted that rubber is economically significant, but a massive amount of rubber waste is observed in the environment scarcely degraded, leading to waste accumulation [8]. After curing elastomers at a high temperature level, a crosslinked structure with improved mechanical features is generated by sulfur linkage connecting the carbons of main rubber chains [9]. Vulcanization generates sulfur crosslinks (a three-dimensional network such as monosulfide, disulfide, and polysulfide bonds), changing the non-elastic and sticky materials to elastic and non-sticky ones. As the structure of tires is stable and three-dimensional with stabilizers and additives, rubber waste takes a long time to be degraded [10].

Commonly used approaches, including burning and burying rubber material waste, can harm the climate and pose pollution by releasing poisonous materials like carbon oxide [11]. The pools created due to the accumulation of stagnant rainwater in the curvy structures of disposed rubber waste release toxic substances. It has been found that such substances adversely affect organisms living in marine regions because these compounds can be toxic to aquatic life. In addition, the structures formed by rubber waste may become breeding grounds for rodents and mosquitoes, promoting the proliferation of insects capable of carrying diseases, which impose health risks on human communities. Additionally, a number of harmful compounds are released as a result of rubber degradation, e.g., chemical additives, heavy metals, and long-lasting organic contaminants, e.g., anti-ozonants such as N-(1,3-dimethylbutyl)-N′-phenyl-p-phenylenediamine (6PPD) as well as its toxic transformation product, i.e., 6PPD-quinone. Such substances may penetrate into neighboring ecosystems and enter waterways via runoff and lead to persistent harm to aquatic life and the broader surrounding environment. Even very low concentrations of such toxicant materials may lead to disrupted aquatic life, causing ecological imbalances and additional environmental hazards resulting from discarded rubber waste. The presence of 6PPD and its toxic transformation product, 6PPD-quinone, in environmental runoff has been shown to cause the death of fish, including coho salmon [9,12,13,14,15].

From the statistical data, it was found that for the 32 countries evaluated, which include the United Kingdom, EU27, and Turkey, Norway, Serbia, and Switzerland, the most applied techniques for managing waste tires can be categorized as follows: civil engineering (3%), unknown/stocks (5%), energy recovery (40%), and recycling (52%). However, two special important aspects must be considered during data analysis. Firstly, energy recovery includes the materials’ combustion, and therefore, the potential waste production of the process in solid and volatile pollution forms which should be gathered and handled by appropriate treatment. Also, this technique leads to the potential of low-quality waste tire rubber, which can be a replacement for raw matrix, so the demand for use from natural sources grows. Additionally, widespread recycling is legitimated and supported due to higher societal awareness [16].

There is a kind of recycling called devulcanization: cleaving crosslinked rubber chain bonds [17]. The process by which rubber waste is converted into its primary uncured form through the selective scission of covalent crosslinks within the elastomer matrix is called devulcanization. Ideally, the yield of devulcanization would be a mixture of uncured rubber prepared to be vulcanized with intact polymer chains. Nonetheless, devulcanization is practically concurrent with unwanted side reactions, e.g., oxidation and chain degradation, which weaken the mechanical characteristics of the obtained devulcanized rubber. In addition, the type of curatives significantly affects rubber devulcanization. Given that the bond energies of S–S and C–S bonds are lower than that of C–C covalent bonds, peroxide-cured rubbers are more inclined to participate in side reactions (that is, degradation) in the course of devulcanization compared to sulfur-cured rubbers. Even though the reactions taking place in the course of devulcanization are not clearly understood, the generally accepted main pathways include radical formation, substitution, oxidation, and rearrangement. Even though devulcanization promoting chemicals may lead to triggering of further mechanisms, the application of the devulcanizates is limited by them [18]. Apart from the long history behind devulcanization, particularly over the past ten years, this procedure has gained far more attention from researchers since public concerns have arisen about waste rubber management [19]. In recent years, various rubber recycling techniques have been documented in scientific literature, with a significant focus on mechanical methods (32%), thermo–chemo–mechanical approaches (6%), ultrasonic techniques (4%), chemical methods (19.2%), microwave processes (25.8%), cryogenic methods (1.2%), microbial strategies (2.7%), and integrated thermo–chemo-mechanical and microwave approaches (9.9%), highlighting the use of sustainable technologies and methods [20]. This paper focuses on microwave and biological devulcanization to compare these methods in the technical literature to shed light on the advantages and disadvantages of these procedures.

### 1.1. Literature Review Statistics

This part outlines the major trends in biological desulfurization and microwave devulcanization, emphasizing important points derived from reviewing published papers in the SCOPUS database.

#### 1.1.1. Publication Date

**Figure 1:**
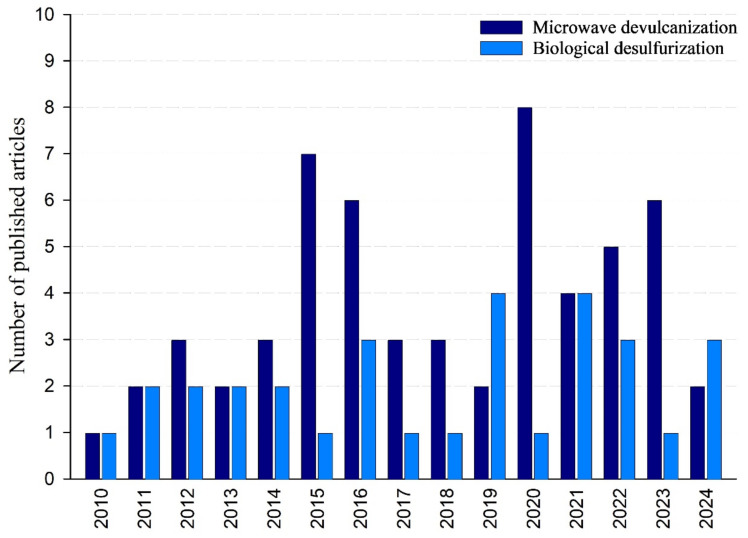
Number of published articles since 2010 to 2024.

Research on microwave devulcanization has gradually increased, with the earliest studies published in 1978. The highest number of published papers reached eight in 2020, and the output has remained fairly high since then, with six papers released in 2023. From 2010 to 2024, this area’s cumulative number of publications amounts to 57 papers. The data indicate a consistent growth in interest, especially in recent years, highlighting an increasing acknowledgment of this devulcanization technique. In comparison, biological desulfurization has experienced more erratic development. The peak number of publications was in 2019 and 2021, with four papers released each year. Overall, 31 papers have been published on this topic from 2010 to 2024. This indicates a more specialized, yet still significant, area of research. Figure 1 illustrates the number of papers issued on biological desulfurization and microwave devulcanization between 2010 and 2024.

#### 1.1.2. Journals

**Figure 2:**
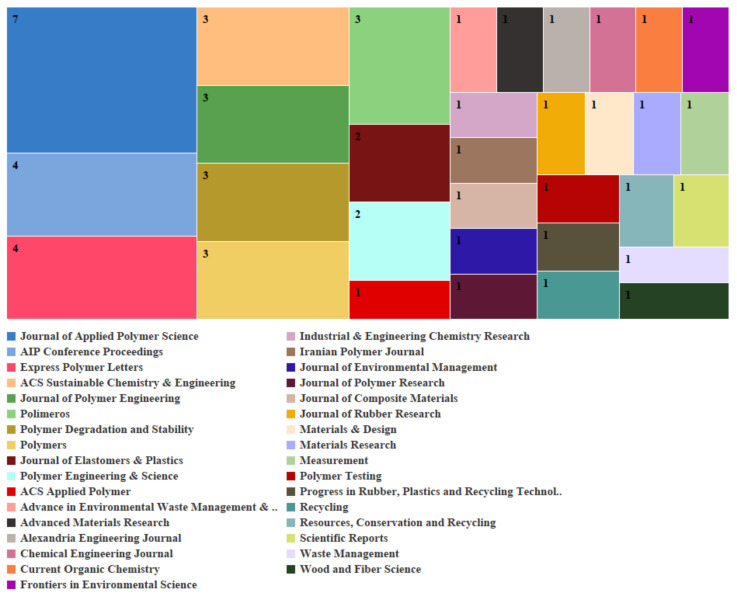
Journals’ published documents related to microwave devulcanization.

**Figure 3:**
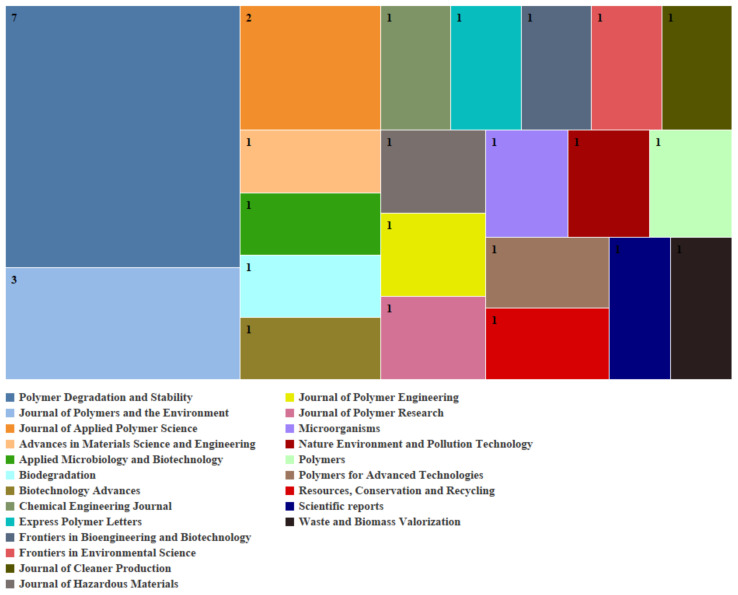
Journals’ published documents related to biological desulfurization.

The Journal of Applied Polymer Science is the leading publication in microwave devulcanization, having released seven papers on the subject. Other important journals comprise the Express Polymer Letters and AIP Conference Proceedings, each contributing four papers. Journals such as Polymers and Polímeros have each published three papers showing the importance of polymer science. The most prominent journal for biological desulfurization is Polymer Degradation and Stability, which has published seven related papers. The other significant journal is the Journal of Polymers and the Environment, which released three papers revealing this field’s environmental emphasis. Journals like Applied Microbiology and Biotechnology, as well as Biotechnology Advances, also hold prominence, reinforcing the biological element of the desulfurization method. Figure 2 and Figure 3 illustrate the various journals that publish research on biological desulfurization and microwave devulcanization.

#### 1.1.3. Subject Areas

**Figure 4:**
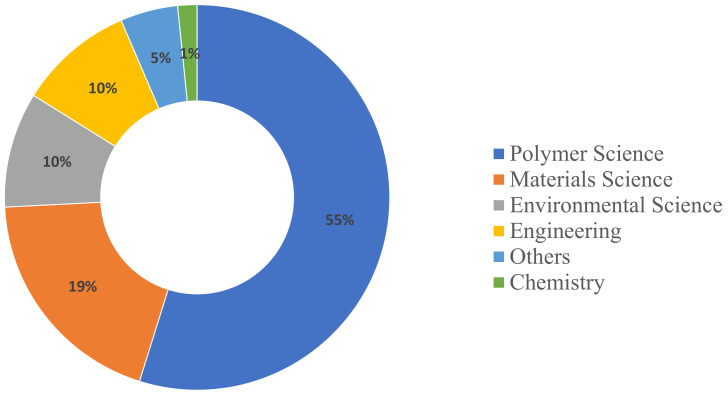
Subject areas related to microwave devulcanization.

**Figure 5:**
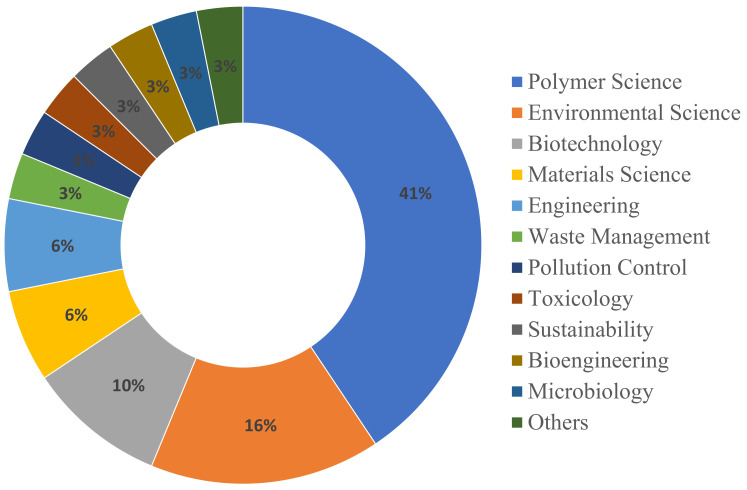
Subject areas related to biology desulfurization.

In microwave devulcanization, polymer science is the primary subject area, with 34 papers highlighting its critical role in innovating materials and improving recycling technologies. Materials Science is also significant, contributing twelve papers, while Engineering and Environmental Science each add six to the discourse. In biological desulfurization, polymer science leads with a total of 13 papers. Trailing behind is Environmental Science, which has five papers underscoring the method’s focus on ecological advantages and sustainability. The dispersal of subject areas for biological desulfurization and microwave devulcanization is depicted in Figure 4 and Figure 5.

#### 1.1.4. Funding Organizations

In microwave devulcanization, the most funded publications originate from Brazilian institutions, mainly the National Council for Scientific and Technological Development (CNPq) and São Paulo Research Foundation (FAPESP), each supporting 11 papers. The leading funding organization for biological desulfurization is the National Natural Science Foundation of China, which has backed nine papers. Other significant funding agencies include the Fondazione Silvio Tronchetti-Provera, with two papers, and the Nature Science Foundation of Beijing, with six papers. Table 1 lists funding organizations with four or more publications in the fields of microwave devulcanization and biological desulfurization.

#### 1.1.5. Countries

Brazil has made a significant contribution to microwave devulcanization research, with a total of 20 publications. Iran and Spain follow closely, each contributing six articles. In the field of biological desulfurization, China has published 14 studies, accounting for nearly half of the global publications on the topic.

As a leading tire producer, Brazil faces the problem of a substantially challenging volume of waste tires. This country produces about 800,000 tons of ELTs annually [21]. The tire production in Brazil noticeably outbalances its attempts at tire disposal, and the evaluated recycling rate only amounts to 10% [22]. Typically, such a large volume of tire waste is utilized as a heating fuel in cement kilns and for different industrial purposes [23]. In spite of the growing volume of waste tires, 20 publications about microwave devulcanization in Brazil have reflected its continued research attempts to enhance the techniques of tire recycling. By enacting a number of laws and regulations, e.g., the Normative Instruction N°001/2010 and CONAMA’s Resolution, the Brazilian government aims to promote appropriate disposal and recycling of ELTs [24]. Therefore, the government is interested in technologies, such as microwave devulcanization, in order to reduce the environmental effects of tire waste. Nonetheless, a number of challenges, e.g., funding problems and weak infrastructures, still prevent the widespread adoption of such solutions [25].

The largest producer of waste tires in the world is China, facing the same challenge [23]. China produces over 13 million tons of waste tires every year, while the recycling rate of the country is about 30% [25]. Therefore, China has resorted to different methods of waste tire management, such as biological desulfurization, in which microorganisms are utilized to break down the sulfur compounds found in the rubber formula. This is because desulfurization improves the recycling efficiency. A number of 14 publications about this subject in China constitute nearly 50% of the entire literature on the subject of biological desulfurization. This indicates that China is very interested in the improvement of its recycling processes for waste tires. Given that it is estimated that China generated 14.58 million tons of waste tires in 2018 alone, the country’s tire production has experienced rapid growth [23]. In spite of the mentioned challenges, biological desulfurization is an encouraging technique to increase tire recycling efficiency. Nonetheless, a number of factors, e.g., inadequate government support and insufficient regulations, have decreased China’s recycling rate, which could be higher in the case of resolving these limitations [25].

In direct response to their large waste generation and tire production, China and Brazil have made considerable contributions to the studies on biological desulfurization and microwave devulcanization, respectively. Given that Brazil is challenged by high volumes of waste tires, often used as fuel and substantial tire production [23], the country has resorted to microwave devulcanization as a technique to enhance recycling approaches. In the same way, given the vast tire waste in China due to the rapid increase in automotive ownership, it has invested in biological desulfurization as a realizable answer to the environmental challenges resulting from tire disposal [26]. To address the global challenge of tire waste, conducting studies in these areas is critical, and both China’s and Brazil’s attempts indicate the relationship between waste management, scientific innovation, and tire production. The increasing volume of studies in the above fields is a direct response to the necessity of more sustainable techniques for waste tire recycling, with both Brazil and China realizing the economic and environmental significance of enhanced recycling technologies.

To summarize, microwave devulcanization has experienced significant growth, especially in Brazil, where substantial financial support has come from the National Council for Scientific and Technological Development (CNPq) and the São Paulo Research Foundation (FAPESP). This study area is heavily oriented towards polymer science, and the Journal of Applied Polymer Science serves as a prominent publication outlet. In biological desulfurization, China stands out as the top country in funding and research publications, with its National Natural Science Foundation being the main organization of financial backing. The research strongly emphasizes polymer science and environmental science, and Polymer Degradation and Stability ranks as the leading journal in this domain.

## 2. Microwave Devulcanization

Microwaves are part of the electromagnetic spectrum, characterized by wavelengths ranging from 1 mm to 1 m and frequencies between 300 MHz and 30 GHz. In microwave heating applications, two frequencies set by the Federal Communications Commission (FCC) for medical, industrial, and scientific uses are typically utilized: 0.195 GHz and 2.45 GHz [27]. In rubber devulcanization at high temperatures, using microwave energy is a promising and eco-friendly method worldwide compared to other existing techniques [9]. In the late 1970s, Novotny et al. [28] from The Goodyear Tire & Rubber Company, for the first time, proposed microwaves for waste rubber devulcanization [29]. This method can be easily regulated, allowing the microwave energy to specifically break the sulfur–sulfur (S-S) and carbon–sulfur (C-S) bonds in the crosslinks of sulfur-cured rubbers [27]. A significant challenge faced during the devulcanization of ground tire rubber (GTR) is achieving general performance and efficiency. Vulcanization leads to the formation of a crosslinked framework among the elastomer polymer chains. The crosslinks are made up of –S–S– connections. Many devulcanization techniques tend to not only disrupt the di- or polysulfide bonds but also break the elastomer macromolecules when applied to a specimen. This leads to a reduction in the degree of crosslinking and polymer degradation [20]. Additional investigations conducted on the GTR microwave devulcanization indicated that controlling the processing parameters and temperature is important in order to promote the bond cleaving selectivity and obtain high-quality devulcanizates [30]. One may regard this process as controlled degradation. Thus, the parameters should be adjusted carefully to minimize or prevent the occurrence of chain scission in the polymer backbone [31].

In the microwave-based recycling method, the targeted rubber samples are devulcanized using electromagnetic waves by which the three-dimensional (3D) elastomer network bonds are broken. The microwave heating process of material results from interactions with the electromagnetic field at a molecular level [2]. The energy for heating rubber assists sulfur crosslinks with cleavage. Nevertheless, given non-polar elastomers like tire rubber, microwave energy is scattered and less absorbed, necessitating adding particles like carbon black for energy absorption. This filler can absorb electromagnetic waves using Maxwell–Wagner polarization, enabling the devulcanization process [13]. Over the microwave treatment procedure, the temperature rapidly increases to 260−350 °C [32,33]. The microwave heating process depends on microwave electrical field oscillation interactions with dipole molecules and/or charged ions. Three major mechanisms engage in microwave heating: (1) dipolar polarization, (2) ionic conduction, and (3) interfacial polarization. Dipolar polarization, also called dielectric heating, refers to dipole heating. Ionic conduction is related to heating samples containing ionic species or free ions. Interfacial polarization, known as the Maxwell–Wagner effect, occurs in non-homogeneous samples with conducting particles in non-conducting media [12]. Tangent loss is the significant factor-making material susceptible to being heated through microwaves, as shown in Equation (1) [14,34]:

(1) $$Tan\;\delta =\frac{\varepsilon^{\prime \prime }}{\varepsilon^{\prime }}$$

where *δ* is the dielectric loss, *ε*′, also called the dielectric constant, which refers to the real part of the complex permittivity, indicative of the amounts of electric energy stored in the material heated. *ε*″, also referred to as dielectric loss factor, the imaginary part of the complex permittivity, shows the material’s capacity to absorb microwave energy and convert it into heat [35]. Tan *δ*, the loss tangent, is frequently used to characterize the dielectric response and reflects the material’s efficiency in transforming absorbed energy into heat [36]. Table 2 represents the microwave properties of natural rubber and carbon black powder in terms of *ε*′, *ε*″, and tan *δ*.

As shown in Table 2, the electrical energy stored in the natural rubbers is 2.2 units, of which only 0.01 units can be depleted, i.e., the efficiency of the rubber in converting the absorbed energy into heat is 0.00454. Meanwhile, carbon black stores 10.45 units of electrical energy (4.75 times that of rubber), of which 3.75 units can be depleted (375 times that of natural rubber). Moreover, the efficiency of carbon black in converting the absorbed energy to heat is 0.35855, which is 78.97 times that of natural rubber. This indicates that carbon black is a considerably stronger microwave absorber than natural rubber [35]. Consequently, microwave devulcanization is more effective with polar rubbers, polar-filler rubbers, or fillers capable of absorbing microwaves, like carbon black [30].

**Figure 6:**
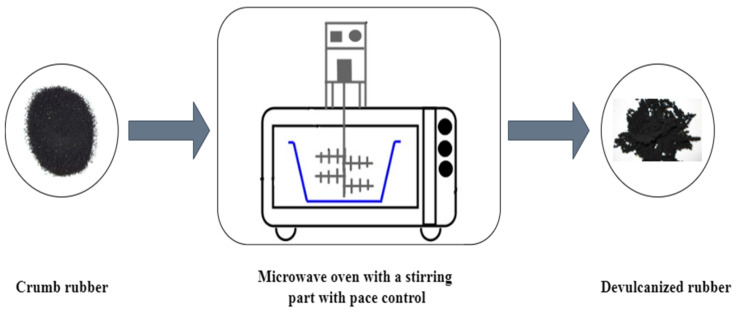
A schematic presentation of the rubber microwave devulcanization system.

Figure 6 schematically depicts the rubber microwave devulcanization system. This figure shows that a commonly used microwave oven equipped with a stirring part with a pace control was used to devulcanize rubber [37]. This motorized polytetrafluoroethylene (PTFE) stirrer was placed in the microwave to expose ground tire rubber (GTR) to the microwaves homogeneously [9,38]. Over the process of devulcanization, a 15–80 g sample of each type of GTR was put in the PTFE (300 mL) or a glass beaker (600 mL) [9,39]. Finally, the sample was exposed to microwave radiation at different exposure times.

The advantages and disadvantages of microwave devulcanization are summarized in Table 3.

### 2.1. Variable Parameters of Microwave Devulcanization

There are several parameters that can affect microwave devulcanization, including exposure time, temperature and heating rate, the particle size of crumb rubbers, stirring speed, NR to SBR ratio, carbon black content, use of devulcanizing reagents, use of ionic liquid, power of microwave oven, and content and type of oil. This section briefly describes each parameter and its effect on the devulcanization process.

#### 2.1.1. Exposure Time

de Sousa et al. used a commonly used microwave oven, as shown in Figure 1, to devulcanize GTR. The exposure time of the material to microwaves was from 1 to 5.5 min. It was confirmed that the temperature rose as the sample exposure time to microwaves increased. Moreover, the figure for the gel fraction and the density of the sample’s crosslinking witnessed a decrease while the GTR exposure time to microwaves increased [40]. In a different investigation by the same author, a prototype microwave oven was utilized to devulcanize natural rubber (NR). The material was exposed to microwaves for 2 to 5 min. In practice, it was found that the gel content diminished as the NR exposure time increased. The final temperature achieved by the specimen was deduced to be a key factor in the devulcanization degree, which was influenced by the exposure duration. Evidently, the devulcanization degree generally increases with longer NR exposure time [34].

Colom et al. investigated the phenomena engaging in GTR microwave devulcanization [9]. The samples were treated by the microwave for 0, 3, and 5 min. It was indicated that temperature increment was caused by the friction of GTR polar components and that the elastomeric macromolecule induced dipole, oxidation, and CO_2_ production, with exposure to the treatment causing a decrease in the amount of carbon black over time.

In Vahdatbin et al.’s study [39], crumb rubber (CR) was treated in a modified domestic microwave oven using a stirring system capable of controlling speed. CR samples were subjected to microwave irradiation for 20, 40, 60, 80, and 100 s. Being subjected to microwave irradiation for 100 s led to the destruction and smoldering of the CR samples. The results indicated that the sol fraction and devulcanization percentage of samples elevated up to 60 s of microwave irradiation and then reduced. This reduction was due to the post-curing of samples as a result of excessive heat generated during the devulcanization process at greater radiation times (i.e., recombining radically made chains generated during microwave irradiation).

Bani et al. introduced a microwave (MW)-induced thermal treatment to achieve the partial devulcanization of a poly-ethylene–propylene–diene monomer (EPDM) rubber infused with carbon black. In fact, the exposure duration varied from five to six to seven minutes. The results indicated a noticeable pattern of temperature increase with more extended exposure times. In this regard, at five minutes, the temperature reached 320 °C; after six minutes, it increased to 340 °C, and at seven minutes, it peaked at 367 °C. This shows that prolonged exposure to microwaves leads to higher temperature levels. The moderate gel content was below 50% in all the experiments conducted. This indicated a notable decrease in crosslink density. They deduced that effective devulcanization processes appeared to commence only at temperatures exceeding 300 °C. Furthermore, measuring the sulfur content and the distribution of crosslinks in both treated and untreated rubber indicated that the treatment greatly influenced them [47].

Raslan et al. utilized a system that involved a prototype national microwave oven, which was modified to include a motorized-speed-control moving approach. The specimens were subjected to microwaves several times for three, five, six, and seven minutes. In general, it was found that increasing the time of microwave exposure leads to a maximum waste tire rubber (WR)temperature following microwave treatment while simultaneously decreasing the gel content in the specimen. This settles a more effective decomposition of the three-dimensional network present in WR. The findings indicate that extended microwave exposure durations result in reduced gel content. Further, the specimen was burned at seven minutes. Ultimately, it is clear that microwave devulcanization at six minutes yields the highest devulcanization percentage, with a soluble fraction of 36% [48].

#### 2.1.2. Temperature and Heating Rate

Simon et al. performed the devulcanization process of ground tire rubber (GTR) using microwaves at various temperatures (140−200 °C) and heating rates (2−18 °C/min). They represented the devulcanized GTR (dGTR) specimens using swelling and Soxhlet extraction tests to assess their crosslink density and soluble content. They conducted Horikx’s analysis. They evaluated the typical microwave energy and selectivity parameters of the specimens. It was evident that using GTR devulcanized at decreased temperatures (140−160 °C) resulted in very high selectivity, with the devulcanization degree being satisfactory (approximately 50 to 70%). When temperatures were raised (180−200 °C), the devulcanization degree increased to 85%, but the selectivity dropped. This shows extreme decomposition in the temperature range. At reduced heating rates and lower temperatures, which means longer residence times, the devulcanization degree improved. However, the heating rate did not significantly impact the devulcanization degree at higher temperatures. A sufficient devulcanization degree and increased selectivity can be reached at temperatures lower than the NR decomposition temperature. When approaching, reaching, or exceeding the NR decomposition temperature, decomposition occurs, and longer residence times result in greater decomposition. The selectivity and devulcanization degree should be considered together. Therefore, they present the K.D number. This value can be determined by multiplying the relative reduction in crosslink density by selectivity. The specimens were evaluated according to their K.D values. Comparing these findings with the typical microwave energy indicated that decomposition is not influenced by the typical microwave energy. Instead, it is solely dependent on temperature and the rate of heating [49].

#### 2.1.3. Particle Size of Crumb Rubber

The control of devulcanization temperature and efficiency was significantly influenced by waste powder particle size. Microwave irradiation produced hot spots in the sample for large particles, i.e., particles larger than 1 cm in diameter, and part of the sample completely deteriorated, but rubber waste with a 1 mm particle size was still able to undergo devulcanization efficiently and effortlessly. With microwave heating, changes in temperature do not take long to appear because volumetric heating is generated from within the material to its surface. Decreasing the waste powder’s particle size made it easier to heat up and devulcanize the rubber, resulting in increased efficiency [50].

The impact of CRs’ size on the devulcanization process was examined by Vahdatbin and Jamshidi [39]. The findings demonstrated that decreasing the particle size resulted in a higher percentage of devulcanization, since a reduction in particle size led to a decrease of the temperature gradient between the surface and bulk of the particles. Consequently, the temperature rises in bulk, generating a finer devulcanization process. Put differently, it could be argued that particle size reduction led to the partial devulcanization of devulcanized crumb rubber samples and expanded the devulcanization process to the particle depth. It was found that the optimum size of crumb rubber particles is 595 µm (mesh number 30).

#### 2.1.4. Stirring Speed

Raising the stirring speed from 0 to 90 rpm resulted in a rise in the sol fraction, which was caused by the consistent surface temperature of the samples at higher stirring speeds. The greatest devulcanization percentage was achieved at a stirring speed of 30 rpm [39].

#### 2.1.5. NR to SBR Ratio

Vahdatbin et al. [39] developed rubber compounds at various NR to SBR ratios (40:60, 50:50, 60:40, 70:30, and 80:20). The findings indicated that increasing the NR–SBR ratio of the samples led to a decrease in the sol fraction, which was attributed to a more effective response of SBR to devulcanization assisted by microwaves. The lesser molecular weight was responsible for the greater instability of SBR polymer chains under microwave irradiation. Findings revealed that the devulcanization percentage reduced when the NR to SBR ratio decreased, indicating a better SBR response to microwave-assisted devulcanization.

#### 2.1.6. Carbon Black Content

One of the materials commonly used in commercial rubber formulas is carbon black (CB), the role of which has been investigated by different authors. Hirayama et al. [33] explored the effect of CB contents on the devulcanization of the ground previously vulcanized SBR. The CB contents of the SBR were from 0 to 100 parts per hundred rubber (phr). The results showed that the vulcanized rubber gel content decreases while the quantity of CB in the rubber rises. Further, SBR devulcanization depends on the amounts of CB within the rubber. The main parameter leading to devulcanization is CB, which heats the material. This behavior is verified when the rubber-related temperature increases dependent on the content of carbon black in the material. This attitude refers to the dielectric constant of CB associated with thermal conductivity with higher values than SBR; it is inferred that the CB absorbs microwave radiation, heats the material, and cleaves sulfur crosslinks.

de Sousa et al. [34] utilized a prototype microwave oven by incorporating a motorized stirring mechanism with adjustable speed to facilitate the devulcanization of natural rubber (NR). The CB content in the samples varied at 0, 20, 45, 60, and 80 phr. It was proved that the NR temperature tended to rise after treatment as the CB content in the rubber rose. As CB acts as a conductive filler, it can receive microwave radiation and transform it into heat. Consequently, greater microwave energy is received by augmenting the quantity of CB in the natural rubber (NR). Generally, the devulcanization degree tends to rise in relation to the CB content incorporated in the rubber.

#### 2.1.7. Use of Devulcanizing Reagents

Khavarnia et al. [51] evaluated the possible impact of multiple devulcanizing reagents such as hexadecylamine (HDA), diphenyl disulfide (DPDS), N-cyclohexyl-2-benzothiozyl sulfenamide (CBS), and tetramethylthiuram disulfide (TMTD) on the process of devulcanization. It was demonstrated that HAD (6 phr) managed to be deemed an appropriate devulcanizing reagent. The devulcanization mechanism of devulcanizing reagents was also proposed. Considering the devulcanization mechanism of HDA, microwave radiation and HDA affect polymer chains, producing two ions through the sulfur crosslinks.

Molanorouzi et al. [52] (like Khavarnia et al. [51]) evaluated how HDA, DPDS, CBS, and TMTD can affect waste tire rubber devulcanization. The results from the Horikx analysis indicated that the DPDS (6 phr)-formulated compound was the most appropriate compound for the process of devulcanization compared to the ones formulated with other mentioned devulcanizing agents. Regarding the action mechanisms of DPDS, CBS, and TMTD, two radicals are produced from polymer chains through sulfur bridges following microwave radiation. Then, the reagent radicals (generated by microwave radiation) and the formed radicals are combined, leading to stable molecules.

Raslan et al. combined the waste rubber (WR) powder with a fixed proportion of 2 phr TMTD and 10 phr spindle oil. Adding oil also causes the rubber to swell and increases the spacing between the rubber chains. This, in turn, makes the crosslinks more accessible to the devulcanizing chemical agents and enhances the effectiveness of devulcanization. The application of microwave irradiation to WR containing TMTD leads to the rapid cleavage of polymer crosslink bonds and the breakdown of TMTD to produce thiocarbamate radicals, which may combine with a degraded polymer radical. This leads to a reduction in molecular weight [48].

A microwave and chemical agents were used by Movahed et al. [50] to devulcanize ethylene–propylene–diene (EPDM) waste rubber. As devulcanizing agents, CBS, dipentamethylenethiuram tetrasulfide (DPTT), HDA, mercaptobenzothiazole (MBT), 2-mercaptobenzothiazole disulfide (MBTS), and TMTD chemicals were utilized in their research. HDA, CBS, and MBT were revealed to devulcanize materials more efficiently than MBST, DPTT, and TMTD. During devulcanization, CBS and MBT were better able to dissolve in aromatic oils due to their aromatic structures, which enabled them to access cleaved rubber radicals more efficiently. As a result of its bigger molecular size, MBTS had less effect than CBS or MBT in cleaving rubber radicals because of a greater steric hindrance. Since TMTD was an aliphatic molecule, the compounds that contained it showed the least amount of devulcanization.

Vahdatbin et al. [39] have applied a novel chemical devulcanizing agent called VitaX in devulcanizing the NR/SBR blend using the microwave irradiation method. Afterward, the impacts of utilizing VitaX and its subsequent content on the samples devulcanized during the intended process were assessed. Results revealed that the approach of adding VitaX significantly impacted the devulcanization percentage by between 18% and 36.45% and exacerbated network breaking, particularly in the crumbed rubbers with small particle sizes.

Paulo et al. [53] investigated microwave-assisted SBR devulcanization by the use of inorganic salts and nitric acid. CuSO_4_, ZnCl_2_, CdCl_2_, and Bi (NO_3_)_3_ (inorganic salts) were dissolved in water (60 mL) and mixed with GTR (25 g) (the particle size was lower than 20 mesh). The substance was then microwaved after being dried for 4 h at 100 °C. The above-mentioned experimental stages were replicated in a second process. Nevertheless, the water (60 mL) used for dissolving inorganic salts was replaced with 25% *v*/*v* nitric acid solution (60 mL). The sum of inorganic salts was measured using the hypothetic stoichiometric reaction between metallic ions and sulfur contained in the rubber, which formed the corresponding sulfide. Owing to the difficulty of dissolving bismuth nitrate inside 60 mL of water, just 25% of the stoichiometric weight was employed. To decrease sulfur crosslinks, GTR was devulcanized by microwave with metallic ions such as cadmium, copper, bismuth, and nitric acid. The ions can react with sulfur atoms to form insoluble sulfide whilst still preventing the creation of new sulfur–carbon bonds in the main polymeric chain. Sulfur–carbon bonds can be attacked by nitric acid, as well. It was reported that only the rubbers comprising copper, zinc, or bismuth had a greater devulcanization level than the ones possessing no metal ions, while the existence of cadmium raises the reticulation value of rubber. This implies the existence of chemical equilibrium, and metal ions might contribute to crosslink formation or sulfur–carbon bond breakage. Once the rubber is only treated by nitric acid, no noticeable difference in microwave-mediated devulcanization is detected. Nevertheless, as acid and metallic ions are mixed, the microwave facilitates free polymeric chain generation throughout the samples.

#### 2.1.8. Use of Ionic Liquid

Seghar et al. [12] examined the impact of an ionic liquid (IL), pyrrolidinium hydrogen sulfate [Pyrr][HSO_4_], as it serves as a proper heat carrier and absorbs microwave energy well, increasing the mixture’s temperature during devulcanization. Therefore, the ionic liquid was used to transfer microwave energy, causing the material core to granulate and the temperature to distribute uniformly through the particles. It was demonstrated that IL positively affects the devulcanization mechanism by microwave. Moreover, while microwave energy increased and SBR was spread through IL, the amount of devulcanization was higher than in SBR without IL. This verifies that IL performs a positive role in devulcanization. Temperature distribution analysis on the ground rubber evidenced that the joint application of microwave energy and IL leads to effective heat production.

#### 2.1.9. Power of the Microwave Oven

Aoudia et al. [32] devulcanized GTR in a home microwave oven. It was discovered that the degree of devulcanization increases as a function of energy used in microwave treatment, up to 1.389 kJ kg^−1^. Further than this level, there is only a slight increase in the devulcanization rate, as it advances asymptotically to 95 percent. These findings imply that a low energy level is sufficient to complete the process of devulcanization or regeneration. Furthermore, the microwave procedure must be optimized, i.e., the appropriate microwave energy must be established, which results in devulcanization with almost no rubber degradation.

#### 2.1.10. Content and Type of Oil

Movahed et al. examined how various amounts of aromatic and aliphatic oils affect the devulcanization of waste powder [50]. Several roles were played by oil during devulcanization. As a radical acceptor, it accelerated oxidation and prevented sol from forming in devulcanized rubber, as well as rising plasticity. Furthermore, oil caused the rubber to swell and the spacing among the rubber chains to grow. Through this process, the crosslinks became more available to the devulcanizing chemical agents (MBTS as the devulcanizing agent), and the devulcanization process became more efficient. Among the compounds with aliphatic oils, the devulcanization percentage was lowest, while that of aromatic oils was highest. Because MBTS has an aromatic structure, it dissolves effortlessly in aromatic oils. Devulcanized rubber chain radicals were recombined with the radicals made by microwaves when the aromatic oil penetrated the rubber matrix.

Khavarnia et al. [51] looked into how the type and content of oil can affect the devulcanization mechanism. The research was conducted with various concentrations of aromatic and paraffinic oils (15, 30, and 45 phr). No impact was seen on the devulcanization percentage after raising the paraffinic oil content from 15 to 30 phr, but it improved the devulcanization mechanism by placing the datasets on a sulfur crosslink cleavage. The excess increment in paraffinic oil negatively impacted the devulcanization procedure. Extra oil can make it difficult for the devulcanization reagent to access macromolecules. Furthermore, comparing the devulcanization percentage of two substances with the same concentrations (30 phr) of aromatic and paraffinic oils indicates that aromatic oil has a detrimental impact on the devulcanization mechanism.

Molanorouzi et al. [52] evaluated the influence of different concentrations of devulcanizing substances and aromatic and paraffinic oils on waste tire powder devulcanization (as did Khavarnia et al. [51]). It was inferred that raising the aromatic oil concentration (15 to 30 phr) had no influence on the devulcanization rate but showed a favorable impact on the devulcanization procedure by placing the sets of data on the curve of a sulfur crosslink cleavage (based on Horix analysis). Raising the aromatic oil concentration to 45 phr indicated a negative impact on the devulcanization mechanism via placing the data points under the curve of the sulfur crosslink cleavage, even though the devulcanization level rose from 35 to 40%. Comparing the devulcanization percentages of two substances with 30 phr aromatic oil and the same quantity of paraffinic oil shows that paraffinic oil negatively influences the devulcanization mechanism. The explanation may be that DPDS is less soluble in paraffinic oil than in aromatic oil. Herein, developed DPDS radicals are unable to access fragmented macromolecules quickly.

Pistor et al. proposed the chips of ethylene–propylene–diene rubber (EPDM-r) from the automotive industry and tested them at various microwave durations (2–5 min). The recycled rubber specimens were analyzed with paraffinic oil in their original state and after oil extraction. The findings indicated that paraffinic oil influences the devulcanization process of EPDM when subjected to microwave treatment. Devulcanization is most effectively managed when paraffinic oil is removed from the rubber mix and specimens are subjected to microwaves for brief intervals (up to 4 min). Elevated peak temperatures were noted in the specimens lacking paraffinic oil because of the higher amount of carbon black and the lack of oil evaporation or decomposition [54].

## 3. Biological Desulfurization

Tire biodegradation refers to how tire materials are broken into simpler compounds by microorganisms. This process is crucial for minimizing the environmental effects of discarded tires, which have the potential to contaminate water, air, and soil with microplastics and dangerous chemicals [44]. In biological recycling, the breakdown of the polymeric structures of solid waste is carried out by living microorganisms, including fungi, algae, and bacteria. To decompose waste rubbers, various biodegrading agents were collected from soil/oceans and cultured. These microorganisms consume sulfur as a key nutrient for their own reproduction and growth, leading to rubber devulcanization [45]. Vulcanized elastomers are resistant to microbial raid. Nonetheless, in specific instances, microorganisms like fungi and bacteria can perform devulcanization by selectively disrupting the S–S bonds. The biological process of desulfurization by microbes is regarded as an environmentally friendly and cost-effective alternative. It provides more selective devulcanization than physical and chemical methods. Despite its several advantages, this novel technique is often not favored by industries due to its slower reactivity. In this approach, up to 4.7% of sulfur can be eliminated during 40 days [44]. Many studies have been conducted in previous decades on ground rubber microbial desulfurization by bacteria and fungi converting sulfur crosslinks to simple sulfur substances or inorganic sulfate [55]. A certain group of fungi, i.e., the white rot fungi, is among the microorganisms with the capability of degrading aromatic structures and has therefore been widely investigated. One of their interesting characteristics is their lignin-degrading enzymes, which make them favorable for a variety of biotechnological purposes, such as bioremediation of soils polluted by organic contaminants and bleaching of kraft pulps [56]. Numerous approaches for bacterial devulcanization on various elastomer latex have been documented in the literature, in both in anaerobic and aerobic settings with various bacterial strands. During anaerobic bacterial devulcanization, bacteria that reduce sulfur are included, whereas in aerobic environments, sulfur generates sulfone groups on the surface of elastomers [44]. In particular, among the CNM (*Corynebacterium*, *Nocardia*, *Mycobacterium*) group, the rubber-degrading strains are the bacteria with the most effective rubber-degrading characteristics among the prokaryotes with the capability of rubber degradation [57]. Typically, the authors noted a reaction time ranging from 1 to 30 days, with a reaction temperature set at 30 °C. They observed a reduction in the sulfur content of the specimen between 8 and 30%. They also obtained a partially devulcanized product that can be revulcanized to produce a material exhibiting identical or enhanced mechanical properties in comparison to virgin rubber [30].

Microorganisms capable of attacking rubber have been identified in the environment with moderate levels of physical factors such as pH, temperature, and salinity. In addition, it is stated that the presence of rubber-cleaving enzymes in the environment is needed to initiate microbial attacks on rubber material [8]. In accordance with previous investigations, based on the oxygen requirement of the bacteria utilized for the devulcanization of rubber, one can classify them into aerobic sulfur-oxidizing bacteria and anaerobic sulfur-reducing bacteria archaea. Besides the condition of their culture, anaerobic archaea outperformed aerobic bacteria in producing high-quality rubbers through the prevention of rubber oxidation [45].

From the performance point of view, bacteria are classified as rubber-degrading microorganisms and rubber-desulfurizing microorganisms, which cleave the C=C bonds of the rubber surface and remove sulfur (S-C and S-S) bonds [58]. In desulfurization, a reaction is performed on the rubber surface, which is approximately 1–2 mm thick. It greatly reduces the quantity of ground rubber sulfur and enhances the mechanical characteristics of natural rubber vulcanizates containing desulfurized ground rubber [55].

Desulfurization by Acidithiobacillus has also been more effective than chemical treatment. Research on *Sphingomonous* sp. revealed S–C and S–S bond breakage in desulfurized rubber. Another report showed that *Alicyclobacillus* sp. causes the largest degree of desulfurization (62.5 percent) [59]. In 1990, Torma and Raghavan [60] performed one of the first studies on GTR biodesulfurization. During this process, *Thiobacillus ferrooxidans* and *T. thiooxidans* were applied to rubber consisting of 15.5 percent sulfur, and eventually, the sulfate concentration was estimated at 350 ppm. Chemolithotroph research was continued until 2011. Li et al. [61] actually discovered that GTR devulcanized with natural rubber (NR) composite materials by *T. ferrooxidans* had stronger mechanical features and lower levels of crosslinks than non-devulcanized GTR (containing NR composites) [62]. GTR desulfurization was conducted in a changed Silverman medium over 30 days while cultivating *T. ferrooxidans*. The rising levels of sulfate ions within the medium evidently showed that the sulfur on the GTR surface was oxidized [44].

Subsequently, Yao et al. chose a microbe, *Alicyclobacillus* sp., which has a desulfurizing ability to recycle waste latex rubber (WLR). They examined the microbial desulfurization activity concerning WLR and investigated the undetermined mechanism involved. *Alicyclobacillus* sp. is affected by the rubber additives present in WLR. Thus, optimizing the WLR concentration is crucial for achieving favorable outcomes. The impact of WLR amounts on *Alicyclobacillus* sp.’s growth was analyzed. The concentrations of WLR in the medium at 2% and 5% (*w*/*v*) indicated optimal microbial growth [63]. Although there is a growing number of studies on rubber biological devulcanization, few companies are currently engaged in the commercial use of this technology on a small to medium scale. For instance, the Recircle group utilizes a bacterial devulcanization method on different kinds of synthetic and natural ground rubber, resulting in an intermediate that is capable of being revulcanized [64]. In brief, microorganisms, such as *Sphingomonas* sp. and *Acidithiobacillus* sp., may contribute to sulfur oxidation by reducing oxygen (aerobic process), which is an exothermic process. In an investigation that used *Acidithiobacillus* in order to treat crumb rubber, desulfurization using microbial solutions was 22% more effective in comparison with the chemical treatment by employing the Neospagnol T-20 technique. *Sphingomonas* sp. has been reported to cause S–S and S–C bond scissions in desulfurized rubber. An investigation using *Alicyclobacillus* sp. reported a noticeable desulfurization (62.5%) [65].

Biotechnological methods indicate many benefits over physicochemical ones, which are usually energy-intensive or involve the use of toxic materials [56]. Employing microorganisms for the recycling of waste rubber has its advantages and disadvantages, as summarized in Table 4.

**Figure 7:**
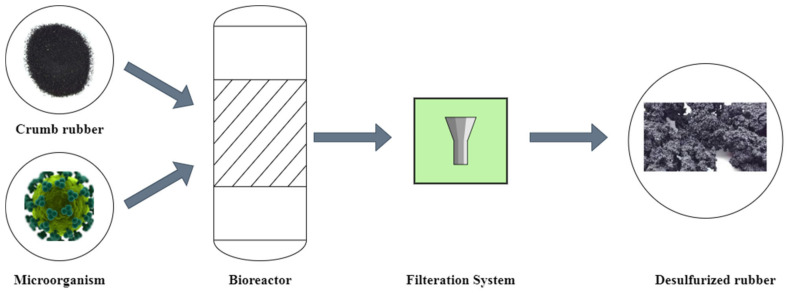
A schematic presentation of the rubber biological desulfurization system.

Microorganisms that desulfurize rubber are often vulnerable to rubber additives. To avoid this issue, the rubber content should be detoxified before recycling. Rubber ethanol leaching has been reported to eliminate poisonous additives [56]. Accordingly, in the desulfurization procedure, the rubber material is first washed with ethanol 96% or chloroform and acetone in using various washing cycles (two–three times) [69]. Following the selection of a strain (bacterial or fungoid), the culture medium with optimized initial pH is required. Based on the literature, the mineral salts medium (MSM) was the most commonly used medium for other bacteria [69]. In flasks filled with the mineral medium, microbial desulfurization was carried out. Crumb rubber (CR) in an amount of 50–150 g was added to the flask [59,62], and the incubation period was set to 8–36 days [59,62]. Desulfurizing microorganisms were found in the soil as well as in waste-activated sludge. Figure 7 schematically shows the rubber biological desulfurization system. As shown in this figure, the flasks were incubated at 34 °C while shaken on a rotary shaker at 170 rpm. The pH was inspected and calibrated to 7, and the medium was added again to replace the liquid sampled [59].

### 3.1. Variable Parameters of Biological Desulfurization

Several parameters can influence biological desulfurization, including microorganism strains, incubation time, rubber percentage in the medium, ground tire mesh size, and surfactant use. This section briefly describes each parameter and its effect on the biological desulfurization process.

#### 3.1.1. Microorganism Strains

Tatangelo et al. [62] studied the properties of microbial species throughout GTR desulfurization by using two bacterial strains, (i) *Gordonia desulfuricans* and (ii) *Rhodococcus* sp. The sample incubated with *G. desulfuricans* displayed a slight increase (from 2.3 to 3.3) and a decline in sol fraction and gel content, respectively, suggesting that this strain may have increased devulcanization activity. Nevertheless, each of the samples had equal sol and gel fraction values. This process is clarified considering the biological desulfurization technique, which is a superficial procedure providing devulcanization only on the surface of GTR and incapable of generating a substantial proportion of sol fraction.

Marchut-Mikołajczyk et al. [72] studied the capability of *Candida methanosorbosa* BP-6 in the degradation of GTR non-treated/treated with ozone. According to their findings, *Candida methanosorbosa* BP-6 strain can decompose GTR non-treated/treated with ozone. Nonetheless, the most noticeable changes were detected in samples undergoing pre-treatment with ozone. Ground tire rubber’s ozonation enhanced biosurfactant production and improved microorganisms’ metabolic conditions. Eventually, one can deduce that [45] even though the metabolic conditions of the *C. methanosorbosa* BP-6 yeast strain were higher in ozonide rubber (increased devulcanization by 20%), the pre-treatment with ozone slowed bacterial growth down.

*Sphingomonas* sp. was used by Li et al. [73] for GTR desulfurization. Following a three-day incubation period, GTR and glucose were applied to the culture medium. They reported that *Sphingomonas* sp. has biological activity against sulfur; thus, it is suitable for desulfurizing GTR and continually grows in the presence of GTR. *Sphingomonas* sp. could break down the conjugated C=C of waste rubber and reduce the amount of sulfur on the GTR surface by 22.9 percent. Sulfur crosslinks could be partially broken after the desulfurization process, leading to the conversion of sulfur to sulfite or sulfur-based groups containing oxygen. Although some GTR crosslinks were broken following desulfurization, the sol fraction remained relatively low since microbial desulfurization showed a surface impact (only a few micrometers into GTR), and the GTR inner sections stayed intact.

Cui et al. [66] desulfurized GTR using *Sphingomonas* sp., *Gordonia* sp., and their combined consortium. *Sphingomonas* sp. and *Gordonia* sp. were cultured in two separate media to determine the best medium for co-culture. Throughout the co-culture desulfurization step, the mixed bacteria biomass was higher than any single bacteria, showing that these two strains of bacteria would stimulate and grow together. The mixed bacteria had the best desulfurization effect, with a desulfurization depth of 6 mm. Ultimately, one can deduce that [45] *Sphingomonas* sp. showed lower contents of oxygen and sulfur by 19.4% and 24.7% compared with GTR, respectively. However, *Gordonia* sp. showed lower contents of oxygen and sulfur by 20.2% and 19.4% compared with GTR, respectively. Finally, both led to 9.5% reduced crosslink density and 6.7% increased swelling value (2.8% and 3.3% more than *Gordonia* sp. and *Sphingomonas* sp.).

Ghavipanjeh et al. [69] used different bacteria (*Thiobacillus*, *Gordonia*, *Nocardia*, *Amycolaptopsis*, and *Pseudomonas*) with the same substrate and under the same environmental conditions for proper comparison to evaluate the biological devulcanization of ground tires (GTs). It was concluded that *Thiobacillus* did not cause an increase in the medium sulfate and a decline in tire matrix sulfur. Nevertheless, after 20 days of incubation, DSMZ 583 and DSMZ 1647 raised the sulfate to around 5% instead of their blank samples and diminished the GT sulfur by 27 and 15%, respectively. Among various strains, DSMZ 44369 effectively raised the amount of sulfate by around 7%. The other ones raised the amount of sulfate in the medium by ca. 1–5%, but for *Thiobacillus* strains. Eventually, one can deduce that [45] the most significant factors in the devulcanization of rubber are rubber size and content, with larger rubber particles paving the way for more devulcanization. In addition, all bacteria in 0.5% *w*/*v* could desulfurize the rubber between 6 and 21%, and *Thiobacillus ferroxidans* PTCC 1647 and DSMZ 583 were the only species that maintained their efficiency in higher rubber contents.

*Thiobacillus* sp. was used by Li et al. [68] for the surface desulfurization (biological) of ground tire rubber. It was concluded that following treatment, oxygen concentration on the waste rubber surface rose by 30%, and sulfur crosslinks were partially broken and then converted to sulfate or sulfur-based groups containing oxygen. S-S bond and S-C bond rations were reduced by 18.3 percent and 42.3 percent each, and S-O bonds were found to form. GTR had a contact angle of 120.5° before treatment, which decreased to 93.5° afterward.

J. K. Kim et al. [70] explored the desulfurization of vulcanized ground rubber by employing two distinct microbial treatments using *T. peromatabolis* and chemical treatment using di-(cobenzanidophenyl) disulfide. According to the experimental results, these treatments enhanced the properties of the final product and the processing of crumb rubber, while the most effective treatment was the microbial treatment. Even though the content of sulfur in the ground rubber was reduced by up to 8% when chemically treated, it was decreased by 30% when undergoing microbial treatment for 30 days. Eventually, one can conclude that [45], in comparison with chemical devulcanization through di-(cobenzanidopheny)-disulfide solvent, biological treatment led to a 20% higher decrease in sulfur bonds during a period of 30 days.

A *Sphingomonas* sp. strain was utilized by Jiang et al. [55] for the desulfurization of SBR ground rubber under optimal cultivation conditions. The sol fraction of SBR ground rubber after *Sphingomonas* sp. desulfurization rose from 4.3% to 7.3%. Despite observing main chain and crosslink breakage, the sol fraction levels were remarkably low in the SBR ground rubber crosslinked network. This happens because microbial desulfurization, as a superficial activity, affects the surface of SBR ground rubber at multiple micrometers deep; the inner part of the SBR ground rubber was unaffected, in any case. The FTIR-ATR and XPS findings demonstrated that C=C, S–C, and S–S bonds underwent breakage in the desulfurized SBR vulcanizates.

Li et al. [61] utilized *Thiobacillus ferrooxidans* in rubber desulfurization after being isolated and cultivated. It was concluded that *T. ferrooxidans* has significantly affected the sulfur element metabolism and the crosslinked S of the GTR surface. Microorganisms managed to cleave conjugated C=C and alter the chemical composition of the GTR surface. Desulfurization partially destroyed the S crosslinked bonds of the GTR surface, resulting in sulfur oxide group formation. The sol fraction rose from 4.69% to 7.43%. In sum, [45] *T. ferrooxidans* resulted in a rise in sol fraction from 4.69% to 7.43% (with only superficial devulcanization), and the mechanical behavior of NR filled with devulcanized GTR (DGTR) improved as a result of enhanced bonding between the two rubbers resulting from the breakdown of interfacial crosslink bonds of devulcanized GTR.

*Alicyclobacillus* sp. was chosen by Yao et al. [63] to desulfurize waste latex rubber (WLR). The findings demonstrated that the sulfur bonds of the waste latex rubber (WLR) surface were disrupted to generate sulfones groups, and the hydrophilic characteristics of desulfurized waste latex rubber (DWLR) were enhanced. *Alicyclobacillus* sp. desulfurized WLR mildly and efficiently. The microorganisms effectively cleaved the sulfur crosslinks in the WLR surface while leaving the main chains unchanged.

Sato et al. [71] demonstrated that a lignin-degrading wood-rotting basidiomycete, *Ceriporiospsis subvermsipora*, specifically devulcanized rubber. They have also shown explicitly that *C. subvermispora*, a wood-rotting basidiomycete, oxidatively broke down sulfide bonds in vulcanized NR sheets to reduce the density of the crosslinked network. The breakage of S-C bonds was also found in the inner portion of the rubber sheets. S-C bonds were cleaved quite preferably by the fungus compared to S-S bonds within polysulfide bonds. Moreover, they show that *D. squalens* cannot break down sulfide bridges in polyisoprene chains but oxidizes unbound sulfides in rubber. *D. squalens*, like *C. subvermispora*, is a potent lignin degrader. These fungi produce lignin-degrading enzymes, including manganese peroxidase (MnP) and laccase (Lac).

#### 3.1.2. Incubation Time

Ghavipanjeh et al. [69] examined the impact of incubation time (10 and 20 days) on desulfurization. After ten days of incubation, there was no discernible difference in the sulfate composition of the media. As a result, for all the microorganisms, the comparative experiment was carried out for up to 20 days of incubation. The findings also showed that a 20-day incubation period is advantageous compared to 10 days for the devulcanization procedure.

Yao et al. [63] examined the impact of desulfurization duration. They realized that in less than five days of desulfurization, the swelling and density of desulfurized waste latex rubber (DWLR) crosslinks vary slightly from those figures for waste latex rubber (WLR). The DWLR swelling grows, and the density of the crosslinks declines during a desulfurization time of about five days. After approximately six days of desulfurization with *Alicyclobacillus* sp., elevated levels of WLR desulfurization could be achieved. In brief, as the desulfurization time grew, the DWLR swelling value was raised while the density of the crosslinks witnessed a decline. After a 10-day co-culture, *Alicyclobacillus* sp. had a strong desulfurization impact on the WLR.

#### 3.1.3. Rubber Percentage in the Medium

Ghavipanjeh et al. [69] studied the impact of desulfurization on the ground tire percentage (0.5 and 5% *w*/*v*) in the medium. This study showed that *Thiobacillus* strains DSMZ 583 and PTCC 1647 had greater efficiency in GT devulcanization at five percent (*w*/*v*) of GTs, compared with *Thiobacillus* strains DSMZ 583 and PTCC 1647 at half a percent (*w*/*v*) of ground tires. This indicates that the microorganisms have a greater quantity of substrate and surface areas to work with, resulting in increased bacterial activity.

Li et al. [68] investigated *Sphingomonas* sp.’s adaptability to SBR ground rubber at concentrations of 0.5 to 4% g/L. It was proved that when the SBR ground rubber volume was 0.5–2% (*w*/*v*), *Sphingomonas* sp.’s biomass declined by around 20–30%; as the concentration of SBR ground rubber increased to 3–4 percent (*w*/*v*), it was reduced 66–78%.

Yao et al. [63] explored the impact of waste latex rubber (WLR) concentration (2–11% *w*/*v*) on *Alicyclobacillus* sp.’s growth. Any rubber additives within WLR are toxic to *Alicyclobacillus* sp. The quantity of WLR can influence *Alicyclobacillus* sp.’s growth. Given the impact of desulfurization and its financial benefits, finding an appropriate amount of WLR to apply to the medium of *Alicyclobacillus* sp. is critical. They reported that when 2% and 5% (*w*/*v*) of WLR were introduced to the medium, *Alicyclobacillus* sp. grew aggressively, as shown by biomass comparable to that of *Alicyclobacillus* sp. continuing to grow on its own. Nevertheless, the germ’s growth was hindered by a biomass decline at 8% (*w*/*v*) WLR or higher. Therefore, 5% (*w*/*v*) WLR appears to be optimal.

#### 3.1.4. Mesh Size of Ground Tire

According to Ghavipanjeh et al. [69], the GT’s quantity and mesh size are major elements in GT biological devulcanization. It should be noted that the large-sized ones (0.354 mm) with a mesh size of 45 showed more effectiveness than the small-sized ones (0.18–0.25 mm with a mesh size of 60–80). Contrary to popular belief, smaller particles will have more intact surfaces for bacteria, thus increasing productivity. Nonetheless, the particle characteristics would cause other mechanical and physical phenomena, including immersing potential and shear stress, which could impact the particles’ mixing ability and lead to sufficient interaction with the microorganisms and the tire particles. This means that tire particles with a size of over 0.25 and less than 0.354 mm are combined easily than those with sizes ranging from 0.18 to 0.25 mm. Smaller particles are light in weight and illustrate a stronger adhesion to the inner surface of the Erlenmeyer.

#### 3.1.5. Use of Surfactant

Yao et al. [63] studied how surfactant (Tween 80) affects *Alicyclobacillus* sp. growth. Due to the lipophilic and hydrophilic nature of latex rubber and *Alicyclobacillus* sp., respectively, Tween 80 was employed to boost the desulfurization impact by increasing the possibility of interactions between WLR and *Alicyclobacillus* sp. It was revealed that *Alicyclobacillus* sp.’s growth was hindered, as shown by a biomass reduction to 27% at the presence of 0.5% Tween 80. The population of microorganisms begins to decrease as the volume of Tween 80 increases. These findings suggest that Tween 80 is potentially toxic to *Alicyclobacillus* sp. and therefore should not be applied to the culture medium of *Alicyclobacillus* sp. separately.

## 4. Life Cycle Assessment (LCA)

A variety of products can be made from ground rubber, which is a valuable raw material. Environmentally, it is less beneficial than other waste management options due to the fact that its manufacturing process emits harmful chemicals and demands a significant amount of energy. The production of ground rubber from scrap tires should be assessed for its environmental impact to provide valuable advice on how to make the process cleaner.

As a method for evaluating a product’s environmental impacts throughout its lifecycle, the Life Cycle Assessment (LCA) is considered effective and thorough. It includes extracting raw materials, manufacturing, producing, transporting, using, and maintaining a product, as well as disposing of its waste [74]. Table 5 summarizes the CO_2_ emissions of different waste tire rubber treatments, the measurement is stated per functional unit, specifically per kilogram of recovered rubber. The following discussion describes treatments in more detail.

The concept of retreading and reuse includes reprocessing products, byproducts, and released waste. The process of recycling includes proper sorting and recycling techniques, as well as the transportation and recovery of materials as a result of recycling. For every kilogram of rubber recycled, 1.2400 kg of CO_2_ are generated. In order to manufacture asphalt, crumb rubber is transported from a crushing plant to an adapted asphalt plant. For modified asphalt, the rubber granules are introduced individually into the liquid asphalt inside the mixer and mixed with gravel in the tumble dryer before being mixed with gravel in the filtration chamber. This process is unlike the traditional asphalt manufacturing process, which combines liquid asphalt with gravel. According to this method, 1 kg of rubber emits 0.667 kg of CO_2_ [75].

Studies regarding flue gas emission levels from tire combustion can be found in the literature, but there are only a few data available. According to Stockwell et al. [79], the emission factor values for burning tires were 2.890 kg CO_2_ per 1 kg rubber under laboratory conditions [76]. It takes approximately 8–10 h to extract tire oil from shredded or whole tires in an illegal extraction process involving kilns and 500–600 °C. A waste tank collects melted tires through pipes, after which the oil is precipitated and then sold illegally as fuel oil. Carbon residues and liquid fuel are illegally disposed of without being recycled. This type of thermolysis process in ELTs produces hazardous emissions that are harmful to soils, water, and plants. It is estimated that 0.329 kg of CO_2_ per 1 kg of rubber is emitted from illegal tire oil extraction [77].

In end-of-life tire pyrolysis, size-reduced tires are burned at high temperatures in a chemical process. Used as fuel and possibly for other purposes, it produces gas, oil, and carbon black. The type or quality of the tire does not matter when it comes to pyrolysis, unlike retreading, devulcanization, or crumb rubber production. Pyrolysis can be used to treat all used tires. The main obstacle to implementing pyrolysis successfully is technical difficulty. A variety of methods can be used to reprocess char by-products, for instance, into low-quality carbon black, which is unsuitable for tire production. Gaseous fractions (hydrogen, methane, and carbon oxides), liquid fractions (water, tar, and oils), and solid residues (char, ash, and metals) are the main products of this process. It has been found that pyrolysis gas (combustion gas) can serve as a fuel to be reintroduced into the pyrolysis process, thus conserving energy. However, this process generates CO_2_, NO_x_, and SO_2_ emissions when it is burned. Pyrolysis gas emissions for 1 kg of rubber are 0.0545 kg CO_2_ [77].

There has been very little research on the environmental impact of the devulcanization process, and most of the studies refer to chemical methods preceding mechanical grinding treatments [78]. A dynamic devulcanization process involves three steps, the first of which is quite similar to a conventional ambient grinding process, producing fine particles around 20 mesh. In the second step, the chemical bonds are broken by devulcanization. The fine rubber obtained from the initial stage is heated between 200 and 220 °C and compressed between 2.0 and 2.3 MPa for less than 3 h, after being blended with specific chemical substances, such as activating and softening agents. As a result of this dynamic devulcanization process, rubber particles are continuously mixed with chemical reactants by a stirrer in a reactor called a “dynamic desulfurization can”. Desulfurization cans are heated using far-infrared heaters or heat transfer oil circulating around them. To achieve the final devulcanized rubber, various methods, including mastication, refinement, and straining, are employed during the refining process. In the case of dynamic devulcanization technology, long process flows require a lot of energy, which subsequently results in a lot of environmental pressure at the energy production stage. Devulcanized rubber produces valuable products that avoid SBR production, and the use of fossil fuels for SBR is thus diminished [77].

A method called thermo-mechanical devulcanization through co-rotating twin screw extruders (cTSE) has been suggested, but in practice, it has not quite been successful. Maris SpA has patented a cTSE-based thermo-mechanical devulcanization process that eliminates devulcanizing agents, supercritical CO_2_, or solvents, with a product that can be reused in a virgin blend without altering its properties. Electricity consumption was found to have the greatest impact. According to the analysis, among all categories, the most significant impact is caused by the energy needed to heat (extruder heaters) and rotate (extruder motors) the screw extruder. As a result of the inventory analysis, it is evident that devulcanization technology is quite simple, and the units’ contributions to its construction over a long period are very diverse. However, the energy required for the process to run is particularly noticeable, which results in the greatest impact. As a general rule, the recycling process of EPDM through thermo-mechanical devulcanization has the highest environmental impact as a result of its electricity usage [78].

Desulfurization tanks are used to perform chemical-assisted devulcanization. The plasticity of rubber powder can be improved by adding plasticizers like pine tar and rosin. In order to sustain a high temperature of 503 K and a pressure of 2.2 MPa, a desulfurization tank utilizes a coal-fired oil furnace. As part of the refinement process, devulcanized rubber is converted into rubber plates. The largest environmental load is caused by devulcanization, where a large amount of coal is burned, generating pollution such as SO_2_, NO_X_, CO_2_, and dust. As a result of the considerable energy consumption and a significant amount of coal burned during this stage, devulcanization has the highest environmental impact, accounting for roughly 66.2%. The refining process is the second-highest contributor at 29.7%, primarily due to electricity consumption. The preparation of rubber powder accounts for approximately 8.06% of the environmental impact [74].

As a result of the analysis, it was found that the main impact of all treatments is high energy consumption. Nevertheless, the global warming potentials and CO_2_ equivalent of each devulcanization process need to be thoroughly studied.

## 5. Comparing Microwave Devulcanization with Biological Desulfurization

There are some similarities shared between these two methods of devulcanization. To begin with, they are both environmentally friendly [14,61] and require initial processing. Microwave devulcanization involves metal particles removal and biological desulfurization necessitates detoxification, which is accomplished through ethanol 96% or chloroform and acetone rinsing [45,69]. Additionally, both methods entail complications, i.e., the disruption of crosslinks all over the sample surface, albeit in slightly different ways. While in biological desulfurization, the disruption is limited to the surface of the rubber, in microwave devulcanization, this is confined to mere peripheral areas of the carbon black, a sound microwave absorbent, due to low microwave absorption by the non-polar elastomers [13,61].

On the other hand, they also differ in quite a few ways. Firstly, unlike biological desulfurization, devulcanization transfers a massive amount of energy to the rubber particles in a short time [41,80] and is also more cost-intensive, which is because of the expensive microwave reactor and other devices involved. However, researchers use cheaper microwave ovens, which may alter the microwave power characteristics. This means that a portion of the energy is wasted through the hole in the Faraday cage and the remaining energy is absorbed by the components of the stirring system [14]. Next, as opposed to biological desulfurization with zero emissions, microwave devulcanization elicits emissions through the degradation of volatile materials [14,58]. Moreover, it requires thorough mixing; otherwise, hot spots develop in the sample, which results in decreased efficiency, and ultimately, sample disruption, contrary to biological desulfurization [14].

However, despite the mentioned facts, some advantages are associated with the devulcanization method. The first relates to non-contact heat transfer in this method, unlike in biological desulfurization, which requires direct contact with rubber particles (desulfurization occurs on the surface of the particles) [41,61]. Secondly, microwave devulcanization has the advantage of continuous processing due to its short start-up and easily adjustable process parameters, whereas biological desulfurization lacks such an advantage because of the difficulty of microbial control [10,41,70,80].

Another point of difference is the size of the particles. The maximum reductions in crosslink density in microwave devulcanization are associated with smaller-sized samples, which is attributable to the surficial hot spots of larger particles formed during microwave irradiation leading to thermal decomposition [39]. In biological desulfurization, the larger tire particles are more effective than the smaller ones. The mixing capability of tire particles with <0.354 mm sizes surpasses that of the finer particles within the 0.18–0.25 mm range. While being lighter, the smaller particles present more sticking affinity to the inner surface of Erlenmeyer [69].

Regarding time and nature, microwave devulcanization needs much less time (30 s [31] to a max. of 7 min [13]) compared with biological desulfurization (8 days [56] to a maximum of 250 days [71]). One explanation might be its physical nature; microwave energy and the heat generated in the sample are consumed to break the crosslinks. This, in turn, transfers a considerable amount of energy to the sample in just a short time, which results in high productivity [41]. In contrast, biological desulfurization is of a chemical nature; it employs sulfur-oxidizing microorganisms for desulfurization. This is a time-consuming process and requires an environment with moderate levels of factors such as pH, temperature, and salinity [8]. Additionally, the process is further prolonged because, despite the prior removal of the additives from the GTR, residual chemicals within detoxicated GTR can be released, inhibiting the expansion of some of the desulfurizing bacteria. Such releases are due to the long-term cultivation of the samples in media [73].

**Figure 8:**
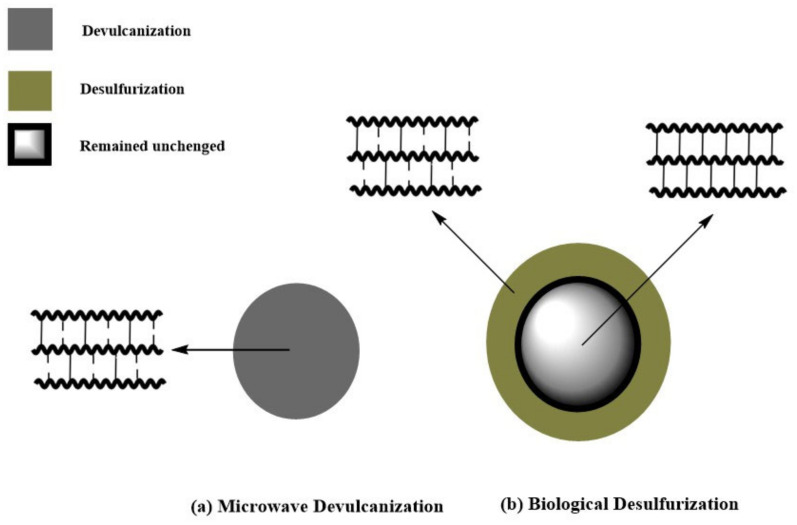
Proposed schematic for efficiency of methods.

Concerning efficiency, devulcanization with microwave benefits from volumetric heating [38], which means that microwave heating occurs in the entire volume of rubber particles [40]. When carbon black—a good microwave absorber—is present, the sample absorbs a considerable amount of energy, which results in the likelihood of crosslink breakage in the entire volume [13,40]. The biological devulcanization procedure is less productive and confined to the surface [66] (a few micrometers deep [68]); the inner sections of the GTR particles stay intact [55,73]. Figure 8 shows the proposed schematic for the efficiency of the methods. This is because microorganisms can only grow in water-based environments, whereas rubber is a material that dissolves in oil [66]. The bacteria’s dimensions do not allow them to permeate into the rubber matrix [62], and the contact surface between ground rubber and the culture medium is seldom compatible [73]. As mentioned earlier, the superficial biological desulfurization procedure can only devulcanize the surface of GTR and thus cannot produce large amounts of sol fraction. That is because culture media and ground rubber show no affinity and the contact surfaces have two separate phases [61]. This merits in-depth scientific investigation [66].

Regarding the need for pre-treatment, unlike microwave devulcanization, biotechnological methods are affected by rubber additives and require prior preparation or detoxification [45], which are disadvantages. The biotechnological processes employ living species that are influenced by the growth environment [56]. One of the problems of such bacterial treatment is the inhabitation of antibacterial activity of anti-aging and curing compounds in rubber [71]. Microorganisms desulfurizing rubber content are often vulnerable to rubber additives [56]. The curing agents tetramethyl thiurame monosulfide (TMTM) and tetramethyl thiurame disulfide (TMTD) and an anti-aging agent, N-(1,3-dimethylbutyl)-N-phenyl-p phenylenediamine (Dusantox 6PPD), have also been shown to prevent the growth of desulfurizing bacteria [71]. To avoid this, the rubber content that would undergo recycling must be detoxified initially. It has been demonstrated that rubber ethanol leaching will eliminate toxic additives, which renders the recycling process environmentally unsustainable [56,71].

Dealing with crosslink density and devulcanization percentage, in biological desulfurization, crosslink breakage occurs only on the surface of the sample [61,66,68], and the crosslinks in the inner part of the sample remain unchanged [55,73]. Therefore, the crosslink density of the sample decreases slightly after desulfurization compared to its crosslink density before desulfurization (Table 6) and causes the devulcanization percentage to have a moderate value (8.84–20.31%). In contrast, in microwave devulcanization, the sample’s entire volume is warmed up by the absorbed waves [40]. Additionally, using a stirrer facilitates the homogeneous distribution of heat [9,39], which means the required energy for breaking the crosslinks will be generated [14,52]. Logically enough, the breakage of crosslinks in the entire volume of the sample is probable, thus improving operation [40]. Moreover, another improvement can be the use of a devulcanization agent [39,51,52] and ionic liquid [12] which improves the breakage of the crosslinks and distribution of the generated heat, respectively. Consequently, the crosslink density of the sample after desulfurization experienced a severe reduction compared to the crosslink density of the sample before devulcanization (Table 6) and the devulcanization percentage can also be enhanced by up to 82.60%.

Regarding operation costs, the microwave devulcanization process demands accurate control of temperature distribution throughout treatment to secure the terminal quality of products obtained. It is a prevalent problem in such technology and can be tackled using hi-tech microwave reactors that are prohibitively expensive. Another solution (generally adopted by academics) is to modify inexpensive domestic-type ovens by adding a stirring system [14]. In contrast, biological desulfurization is economical as it necessitates fewer tools and is less energy-intensive [63,66].

Finally, Table 7 lists the percentage of change in the mechanical properties of rubber containing 10 phr devulcanized rubber. Overall, adding 10 phr devulcanized rubber to the raw rubber lowers the mechanical properties of microwave devulcanization. One of the reasons behind this reduction may be that—although the devulcanization percentage is medium in this method—many unbroken crosslinks, which do not participate in revulcanization, exist in the specimen [39]. Meanwhile, in the biological desulfurization method, the addition of 10 phr desulfurized rubber improves the mechanical properties. This was related to the specimen’s stronger crosslink distribution and interfacial bonding among dispersive ground rubber and raw rubber matrix phases [55].

## 6. Conclusions

This state-of-the-art review presents a comprehensive comparison between devulcanization performed via microwave treatment and biological desulfurization. The two methods differ significantly regarding the quality of the resulting polymer, efficiency, and cost.

Devulcanization by microwave is a physical process in which microwave radiation and the heat produced in the sample are used to break the crosslinks quickly. This physical nature leads to efficient control of process parameters like power and time. On the other hand, since this method takes advantage of volumetric heating, the possibility of crosslink cleavage in the whole volume of the sample gets higher. Consequently, the crosslink density decreases more, and the percentage of devulcanization increases with this method. This method is also not sensitive to rubber additives and requires no detoxification before devulcanization.

However, biological desulfurization shows a chemical nature that makes it difficult to manage conditions using microbial agents. Microorganisms are employed in this method to break the crosslinks. Such microorganisms have a low activity rate, causing the elongation of the time needed for desulfurization. The live organisms involved in this method are sensitive to rubber additives, such as curing agents, so they need to be prepared and detoxified before desulfurization. On the other hand, only the crosslinks at the surface of the sample are broken while the inside part stays unchanged, causing a slight reduction in crosslink density. The devulcanization percentage also increases to the average values.

When comparing the two methods for industrial application, it can be seen that biological desulfurization takes a long time and the crosslinks only break on the surface of the rubber particle. This method is also hard to use in the industry due to the difficulty in controlling the conditions for microbes. While microwave devulcanization occurs rapidly, it benefits from continuous processing and the ability to adjust process parameters easily. Thus, microwave treatment appears capable of being used on an industrial scale.

This study recognizes the absence of a proposed mechanism for desulfurization depth, caused by microorganisms on the surface of the rubber, and the affinity between culture media and ground rubber as major knowledge gaps. Future studies are needed to evaluate the integrity of polymers as well as the fate of tires’ toxic compounds in each method. In addition, studies are needed to identify microorganisms that can retain and neutralize toxic compounds in the tire without negatively affecting the polymer structure. Furthermore, techno-economic analysis for polymers obtained through each microwave and biological approach could facilitate future scale-up and industry implementation strategies.

## Figures and Tables

**Table 1:** Funding organizations and the number of funded publications for microwave devulcanization and biological desulfurization (organizations with four or more publications included).

Method	Name of the Organization	Number of Funded Publications
Microwave devulcanization	National Research, Development and Innovation Office of Hungary	4
	Coordination for the Improvement of Higher Education Personnel of Brazil	8
	São Paulo Research Foundation (Brazil)	11
	National Council for Scientific and Technological Development of Brazil	11
Biological desulfurization	Nature Science Foundation of Beijing (China)	6
	National Natural Science Foundation of China	9

**Table 2:** Microwave properties of natural rubber and carbon black powder [35].

Materials	Material Properties		
	*ε*′	*ε*″	Tan *δ*
Natural rubber (25 °C)	2.2	0.01	0.00454
Carbon black powder (20 *v*%)	10.45	3.75	0.35885

**Table 3:** Advantages and disadvantages of the rubber microwave devulcanization technique.

Advantages	Disadvantages
Solvent-free as well as eco-friendly [14,40];	Hot spots issue [14,30];
Energy transfer (not heat transfer), rapid start-up, and stoppage [41];	Low microwave absorbance of non-polar elastomers, including NR, SBR, EPDM [42];
Material selectivity besides non-contact heating [41];	High cost of professional microwave reactors [14,30];
Conveniently adjustable process parameters, for instance, the power source and treatment time [14,38];	Non-polar polymer chains are almost transparent for microwaves [31];
The handling of large quantities of material through a continuous processing method [43];	Problems with efficient mixing and volatile degradation products emission [14];
Easily adjustable process parameters, such as output power and irradiation time [43,44];	Requires preliminary processing for the removal of metal particles (particularly in waste tires) [45];
The rapid and efficient transfer of large amounts of energy to materials within a short timeframe [14,38,41];	There is a significant risk of thermal runaway [30];
Physical phenomena-related, no need for chemicals [34,40];	This creates a need to function under vacuum conditions and increases the cost of the equipment [30];
Microwaves offer volumetric heating of materials, resulting in more uniform heating compared to traditional heating methods that rely on convection and/or conduction [14,29,44];	
Practicable even in thick types of materials [45];	Due to the action of the microwaves, a number of bonds are formed, while others are broken at the same time, and such chemical modifications contribute to the posterior revulcanization of the same materials [40,46];

**Table 4:** Advantages and disadvantages of rubber biological desulfurization.

Advantages	Disadvantages
Low energy usage levels, easy operation, minimal equipment required, and no pollutants [63,66];	It needs much more time (eight days [56] to a max. of two hundred fifty days [67]) compared to other methods;
No emission of poisonous or hazardous chemicals and not being energy-consuming [58];	Needs initial processing (detoxification) to eliminate toxic additives [45,56];
Only the cleavage of the surface sulfur crosslinks in ground rubber is economical and environmentally safe as opposed to physicochemical methods, which are typically energy-intensive or involve toxic chemicals [68];	The biological desulfurization procedure is capable of devulcanizing only the surface of GTR and thus incapable of producing the sol fraction at high amounts [30,61];
As microorganisms have different desulfurizing enzymes, they can specifically cut sulfur crosslinks on the rubber surface [69];	The control of the required conditions for microbes is difficult [70];
Compared to mechanical and physical techniques, they are more eco-friendly, cost-effective, and selective [61];	Microorganisms employed to desulfurize rubber content are often vulnerable to rubber additives [71];
A number of microorganisms can cleave sulfur crosslinks on the rubber surface while leaving the backbone intact, resulting in recycled rubber with a loose crosslinking network [66];	The devulcanization ratio is low [30];
	There is a possibility of bacterial contamination [30];

**Table 5:** CO_2_ emissions of different waste tire rubber treatments for 1 kg rubber.

Treatment	CO_2_ Emissions (kg)	References
Retreading	1.2400	Ortíz-Rodríguez et al. [75]
Grinding for the production of asphalt	0.6670	Ortíz-Rodríguez et al. [75]
Tire combustion	2.8900	Mentes et al. [76]
Illegal tire oil extraction	0.3290	Li et al. [77]
Pyrolysis	0.0545	Li et al. [77]
Dynamic devulcanization	0.1700	Li et al. [77]
Thermo-mechanical devulcanization	0.4390	Costamagna et al. [78]
Chemicals-assisted devulcanization	0.7650	Li et al. [74]

**Table 6:** Comparing crosslink density and devulcanization percentage of devulcanization by microwave and biological desulfurization.

Type	Crosslink Density (mol/cm^3^)		Devulcanizaton Percentage (%)	**Studied by**
	Before Treatment	After Treatment	$\frac{V_{i}-V_{f}}{V_{I}}$ × 100	
Microwave devulcanization	2.95 × 10^−4^	1.55 × 10^−4^	47.45	[12]
	8.03 × 10^−4^	4.11 × 10^−4^	48.81	[37]
	12.44 × 10^−5^	6.07 × 10^−5^	51.20	[40]
	165	44	73.33	[52]
	69	12	82.60	[51]
Biological desulfurization	7.35 × 10^−5^	6.70 × 10^−5^	8.84	[66]
	6.40 × 10^−5^	5.10 × 10^−5^	20.31	[63]

**Table 7:** Comparing mechanical properties of rubber containing ten parts per hundred rubber (Phr) of desulfurized rubber.

Type	Phr of Devulcanized Rubber	Percentage of Change in the Mechanical Properties				Citation
		Tensile Strength (MPa)	Elongation at Break (%)	Modulus at (MPa)		
				100% of Elongation	300% of Elongation	
Microwave devulcanization	10	−4.61	−7.82	-	-	[68]
	10	−6.90	−2.05	+1.01	−0.07	[39]
Biological desulfurization	10	+10.15	+3.28	0	−0.10	[73]
	10	+1.59	+3.39	-	-	[63]

## References

[1] Karabork F., Akdemir A. (2015). Friction and Wear Behavior of Styrene Butadiene Rubber-Based Composites Reinforced with Microwave-Devulcanized Ground Tire Rubber. J. Appl. Polym. Sci..

[2] Pistor V., Zattera A.J. (2014). Degradation Kinetics of Ethylene Propylene Diene Terpolymer Residues Devulcanized by Microwaves. J. Elastomers Plast..

[3] Antoniou N., Stavropoulos G., Zabaniotou A. (2014). Activation of End of Life Tyres Pyrolytic Char for Enhancing Viability of Pyrolysis—Critical Review, Analysis and Recommendations for a Hybrid Dual System. Renew. Sustain. Energy Rev..

[4] Abbas-Abadi M.S., Kusenberg M., Shirazi H.M., Goshayeshi B., Van Geem K.M. (2022). Towards Full Recyclability of End-of-Life Tires: Challenges and Opportunities. J. Clean. Prod..

[5] Hassan M.R., Rodrigue D. (2024). Application of Waste Tire in Construction: A Road towards Sustainability and Circular Economy. Sustainability.

[6] Moasas A.M., Amin M.N., Khan K., Ahmad W., Al-Hashem M.N.A., Deifalla A.F., Ahmad A. (2022). A Worldwide Development in the Accumulation of Waste Tires and Its Utilization in Concrete as a Sustainable Construction Material: A Review. Case Stud. Constr. Mater..

[7] Thomas B.S., Gupta R.C. (2016). A Comprehensive Review on the Applications of Waste Tire Rubber in Cement Concrete. Renew. Sustain. Energy Rev..

[8] Watcharakul S., Röther W., Birke J., Umsakul K., Hodgson B., Jendrossek D. (2016). Biochemical and Spectroscopic Characterization of Purified Latex Clearing Protein (Lcp) from Newly Isolated Rubber Degrading *Rhodococcus rhodochrous* Strain RPK1 Reveals Novel Properties of Lcp. BMC Microbiol..

[9] Colom X., Faliq A., Formela K., Cañavate J. (2016). FTIR Spectroscopic and Thermogravimetric Characterization of Ground Tyre Rubber Devulcanized by Microwave Treatment. Polym. Test..

[10] Sabzekar M., Pourafshari M., Mohammadmahdi S. (2015). Influence of Process Variables on Chemical Devulcanization of Sulfur-Cured Natural Rubber. Polym. Degrad. Stab..

[11] Liu J., Liu P., Zhang X., Lu P., Zhang X., Zhang M. (2016). Fabrication of Magnetic Rubber Composites by Recycling Waste Rubber Powders via a Microwave-Assisted in Situ Surface Modification and Semi-Devulcanization Process. Chem. Eng. J..

[12] Seghar S., Aït Hocine N., Mittal V., Azem S., Al-Zohbi F., Schmaltz B., Poirot N. (2015). Devulcanization of Styrene Butadiene Rubber by Microwave Energy: Effect of the Presence of Ionic Liquid. Express Polym. Lett..

[13] Garcia P.S., de Sousa F.D.B., de Lima J.A., Cruz S.A., Scuracchio C.H. (2015). Devulcanization of Ground Tire Rubber: Physical and Chemical Changes after Different Microwave Exposure Times. Express Polym. Lett..

[14] Formela K., Hejna A., Zedler Ł., Colom X., Cañavate J. (2019). Microwave Treatment in Waste Rubber Recycling—Recent Advances and Limitations. eXPRESS Polym. Lett..

[15] Tian Z., Zhao H., Peter K.T., Gonzalez M., Wetzel J., Wu C., Hu X., Prat J., Mudrock E., Hettinger R. (2020). A Ubiquitous Tire Rubber–Derived Chemical Induces Acute Mortality in Coho Salmon. Science.

[16] Zedler Ł., Shifeng W., Formela K. (2022). Ground Tire Rubber Functionalization as a Promising Approach for the Production of Sustainable Adsorbents of Environmental Pollutants. Sci. Total Environ..

[17] Kaewpetch B., Prasongsuk S., Poompradub S. (2019). Devulcanization of Natural Rubber Vulcanizates by Bacillus Cereus TISTR 2651. eXPRESS Polym. Lett..

[18] Pirityi D.Z., Bárány T., Pölöskei K. (2024). Recycling of EPDM Rubber via Thermomechanical Devulcanization: Batch and Continuous Operations. Polym. Degrad. Stab..

[19] Vega B., Montero L., Lincoln S., Agulló N., Borrós S. (2008). Control of Vulcanizing/Devulcanizing Behavior of Diphenyl Disulfide with Microwaves as the Heating Source. J. Appl. Polym. Sci..

[20] Colom X., Saeb M.R., Cañavate J. (2024). Microstructural Phenomena in Ground Tire Rubber (GTR) Devulcanized via Combined Thermochemomechanical and Microwave Processes Monitored by FTIR and DTGA Assisted by Other Techniques. Express Polym. Lett..

[21] Bittencourt E.S., Fontes C.H.d.O., Moya Rodriguez J.L., Filho S.Á., Ferreira A.M.S. (2020). Forecasting of the Unknown End-of-Life Tire Flow for Control and Decision Making in Urban Solid Waste Management: A Case Study. Waste Manag. Res..

[22] Bittencourt E.S., de Oliveira Fontes C.H., Rodriguez J.L.M., Filho S.Á., Ferreira A.M.S. (2020). Modeling the Socioeconomic Metabolism of End-of-Life Tires Using Structural Equations: A Brazilian Case Study. Sustainability.

[23] Wang Q.Z., Wang N.N., Tseng M.L., Huang Y.M., Li N.L. (2020). Waste Tire Recycling Assessment: Road Application Potential and Carbon Emissions Reduction Analysis of Crumb Rubber Modified Asphalt in China. J. Clean. Prod..

[24] Padilla L., Díaz Á., Anzules W. (2024). Eco-Management of End-of-Life Tires: Advances and Challenges for the Ecuadorian Case. Waste Manag. Res..

[25] Leong S.Y., Lee S.Y., Koh T.Y., Ang D.T.C. (2023). 4R of Rubber Waste Management: Current and Outlook. J. Mater. Cycles Waste Manag..

[26] Xu J., Yu J., Xu J., Sun C., He W., Huang J., Li G. (2020). High-Value Utilization of Waste Tires: A Review with Focus on Modified Carbon Black from Pyrolysis. Sci. Total Environ..

[27] Zhang T., Asaro L., Gratton M., Aït Hocine N. (2024). An Overview on Waste Rubber Recycling by Microwave Devulcanization. J. Environ. Manage..

[28] Novotny D.S., Marsh R.L., Masters F.C., Tally D.N. (1978). Microwave Devulcanization of Rubber. U.S. Patent.

[29] Asaro L., Gratton M., Seghar S., Hocine N.A. (2018). Recycling of Rubber Wastes by Devulcanization. Resour. Conserv. Recycl..

[30] Dorigato A., Rigotti D., Fredi G. (2023). Recent Advances in the Devulcanization Technologies of Industrially Relevant Sulfur-Vulcanized Elastomers. Adv. Ind. Eng. Polym. Res..

[31] Abel D., Pirityi D., Tamás-bényei P., Bárány T. (2019). Microwave Devulcanization of Ground Tire Rubber and Applicability in SBR Compounds. Appl. Polym. Sci..

[32] Aoudia K., Azem S., Aït Hocine N., Gratton M., Pettarin V., Seghar S. (2017). Recycling of Waste Tire Rubber: Microwave Devulcanization and Incorporation in a Thermoset Resin. Waste Manag..

[33] Hirayama D., Saron C. (2012). Chemical Modifications in Styrene-Butadiene Rubber after Microwave Devulcanization. Ind. Eng. Chem. Res..

[34] de Sousa F.D.B., Scuracchio C.H. (2015). The Role of Carbon Black on Devulcanization of Natural Rubber by Microwaves. Mater. Res..

[35] Mishra R.R., Sharma A.K. (2016). Microwave-Material Interaction Phenomena: Heating Mechanisms, Challenges and Opportunities in Material Processing. Compos. Part A Appl. Sci. Manuf..

[36] Gupta M., Leong E.W.W. (2007). Microwaves and Metals.

[37] de Sousa F.D.B., Zanchet A., Scuracchio C.H. (2017). Influence of Reversion in Compounds Containing Recycled Natural Rubber: In Search of Sustainable Processing. J. Appl. Polym. Sci..

[38] Colom X., Marín-Genescà M., Mujal R., Formela K., Cañavate J. (2018). Structural and Physico-Mechanical Properties of Natural Rubber/GTR Composites Devulcanized by Microwaves: Influence of GTR Source and Irradiation Time. J. Compos. Mater..

[39] Vahdatbin M., Jamshidi M. (2022). Using Chemical Agent in Microwave Assisted Devulcanization of NR/SBR Blends: An Effective Recycling Method. Resour. Conserv. Recycl..

[40] de Sousa F.D.B., Scuracchio C.H., Hu G.H., Hoppe S. (2017). Devulcanization of Waste Tire Rubber by Microwaves. Polym. Degrad. Stab..

[41] Menéndez J.A., Arenillas A., Fidalgo B., Fernández Y., Zubizarreta L., Calvo E.G., Bermúdez J.M. (2010). Microwave Heating Processes Involving Carbon Materials. Fuel Process. Technol..

[42] Simon D.Á., Pirityi D.Z., Bárány T. (2020). Devulcanization of Ground Tire Rubber: Microwave and Thermomechanical Approaches. Sci. Rep..

[43] Zanchet A., Carli L.N., Giovanela M., Brandalise R.N., Crespo J.S. (2012). Use of Styrene Butadiene Rubber Industrial Waste Devulcanized by Microwave in Rubber Composites for Automotive Application. Mater. Des..

[44] Kumar A., Dhanorkar R.J., Mohanty S., Gupta V.K. (2024). Advances in Recycling of Waste Vulcanized Rubber Products via Different Sustainable Approaches. Mater. Adv..

[45] Kazemi M., Faisal S., Fini E.H. (2021). State of the Art in Recycling Waste Thermoplastics and Thermosets and Their Applications in Construction. Resour. Conserv. Recycl..

[46] de Sousa F.D.B., Zanchet A., Scuracchio C.H. (2019). From Devulcanization to Revulcanization: Challenges in Getting Recycled Tire Rubber for Technical Applications. ACS Sustain. Chem. Eng..

[47] Bani A., Polacco G., Gallone G. (2011). Microwave-Induced Devulcanization for Poly(Ethylene–Propylene–Diene) Recycling. Appl. Polym. Sci..

[48] Raslan H.A., Fathy E.S., Abdel Aal S.E. (2023). Thermal Aging and Automotive Oil Effects on the Performance of Electron Beam Irradiated Styrene Butadiene Rubber/Waste and Microwave Devulcanized Rubber Blends. Prog. Rubber Plast. Recycl. Technol..

[49] Simon D.Á., Bárány T. (2023). Microwave Devulcanization of Ground Tire Rubber and Its Improved Utilization in Natural Rubber Compounds. ACS Sustain. Chem. Eng..

[50] Movahed S.O., Ansarifar A., Zohuri G., Ghaneie N., Kermany Y. (2014). Devulcanization of Ethylene–Propylene–Diene Waste Rubber by Microwaves and Chemical Agents. J. Elastomers Plast..

[51] Khavarnia M., Movahed S.O. (2016). Butyl Rubber Reclamation by Combined Microwave Radiation and Chemical Reagents. J. Appl. Polym. Sci..

[52] Molanorouzi M., Mohaved S.O. (2016). Reclaiming Waste Tire Rubber by an Irradiation Technique. Polym. Degrad. Stab..

[53] Paulo G.D., Hirayama D., Saron C. (2012). Microwave Devulcanization of Waste Rubber with Inorganic Salts and Nitric Acid. Adv. Mater. Res..

[54] Pistor V., Scuracchio C.H., Oliveira P.J., Fiorio R., Zattera A.J. (2011). Devulcanization of Ethylene-Propylene-Diene Polymer Residues by Microwave—Influence of the Presence of Paraffinic Oil. Polym. Eng. Sci..

[55] Jiang G., Zhao S., Li W., Luo J., Wang Y. (2010). Microbial Desulfurization of SBR Ground Rubber by Sphingomonas Sp. and Its Utilization as Filler in NR Compounds. Polym. Adv. Technol..

[56] Bredberg K., Andersson B.E., Landfors E., Holst O. (2002). Microbial Detoxification of Waste Rubber Material by Wood-Rotting Fungi. Bioresour. Technol..

[57] Tatangelo V., Mangili I., Caracino P., Anzano M., Najmi Z., Bestetti G., Collina E., Franzetti A., Lasagni M. (2016). Biological Devulcanization of Ground Natural Rubber by Gordonia Desulfuricans DSM 44462T Strain. Appl. Microbiol. Biotechnol..

[58] Hu M., Zhao S., Li C., Wang B., Fu Y., Wang Y. (2016). Biodesulfurization of Vulcanized Rubber by Enzymes Induced from Gordonia Amicalisa. Polym. Degrad. Stab..

[59] Kabir S.F., Zheng R., Delgado A.G., Fini E.H. (2021). Use of Microbially Desulfurized Rubber to Produce Sustainable Rubberized Bitumen. Resour. Conserv. Recycl..

[60] Torma A.E., Raghavan D. Biodesulfurization of Rubber Materials: Proceedings of Bioprocess Engineering Symposium. Proceedings of the American Society of Mechanical Engineers (ASME) Winter Annual Meeting.

[61] Li Y., Zhao S., Wang Y. (2011). Microbial Desulfurization of Ground Tire Rubber by Thiobacillus Ferrooxidans. Polym. Degrad. Stab..

[62] Tatangelo V., Mangili I., Caracino P., Bestetti G., Collina E., Anzano M., Branduardi P., Posteri R., Porro D., Lasagni M. (2018). Microbial Desulfurization of Ground Tire Rubber (GTR): Characterization of Microbial Communities and Rheological and Mechanical Properties of GTR and Natural Rubber Composites (GTR/NR). Polym. Degrad. Stab..

[63] Yao C., Zhao S., Wang Y., Wang B., Wei M., Hu M. (2013). Microbial Desulfurization of Waste Latex Rubber with *Alicyclobacillus* sp.. Polym. Degrad. Stab..

[64] Joseph A., George B., Madhusoodanan K., Alex R. (2015). Current Status of Sulphur Vulcanization and Devulcanization Chemistry: Process of Vulcanization. Rubber Sci..

[65] Kabir S.F., Sundar S.V., Robles A., Miranda E.M., Delgado A.G., Fini E.H. (2024). Microbially Mediated Rubber Recycling to Facilitate the Valorization of Scrap Tires. Polymers.

[66] Cui X., Zhao S., Wang B. (2016). Microbial desulfurization for ground tire rubber by mixed consortium-*Sphingomonas* sp. and *Gordonia* sp.. Polym. Degrad. Stab..

[67] Christiansson M., Stenberg B., Wallenberg L.R., Holst O. (1998). Reduction of Surface Sulphur upon Microbial Devulcanization of Rubber Materials. Biotechnol. Lett..

[68] Li Y., Zhao S., Wang Y. (2012). Improvement of the Properties of Natural Rubber/Ground Tire Rubber Composites through Biological Desulfurization of GTR. J. Polym. Res..

[69] Ghavipanjeh F., Ziaei Z., Mohammad R. (2018). Devulcanization of Ground Tires by Different Strains of Bacteria: Optimization of Culture Condition by Taguchi Method. J. Polym. Environ..

[70] Kim J.K., Park J.W. (1998). Biological and Chemical Desulfurization of Crumb Rubber for the Rubber Compounding. Appl. Polym. Sci..

[71] Sato S., Honda Y., Kuwahara M., Kishimoto H., Yagi N. (2004). Microbial Scission of Sulfide Linkages in Vulcanized Natural Rubber by a White Rot Basidiomycete, *Ceriporiopsis subvermispora*. Biomacromolecules.

[72] Marchut-Mikołajczyk O., Drożdżyński P., Januszewicz B., Domański J. (2019). Degradation of Ozonized Tire Rubber by Aniline—Degrading Candida Methanosorbosa BP6 Strain. J. Hazard. Mater. J..

[73] Li Y., Zhao S., Wang Y. (2012). Microbial Desulfurization of Ground Tire Rubber by *Sphingomonas* Sp.: A Novel Technology for Crumb Rubber Composites. Polym. Environ..

[74] Li W., Wang Q., Jin J., Li S. (2014). A Life Cycle Assessment Case Study of Ground Rubber Production from Scrap Tires. Int. J. Life Cycle Assess..

[75] Ortíz-Rodríguez O.O., Ocampo-Duque W., Duque-Salazar L.I. (2017). Environmental Impact of End-of-Life Tires: Life Cycle Assessment Comparison of Three Scenarios from a Case Study in Valle Del Cauca, Colombia. Energies.

[76] Mentes D., Tóth C.E., Nagy G., Muránszky G., Póliska C. (2022). Investigation of Gaseous and Solid Pollutants Emitted from Waste Tire Combustion at Different Temperatures. Waste Manag..

[77] Li X., Xu H., Gao Y., Tao Y. (2010). Comparison of End-of-Life Tire Treatment Technologies: A Chinese Case Study. Waste Manag..

[78] Costamagna M., Brunella V., Luda M.P., Romagnolli U., Muscato B., Girotto M., Baricco M., Rizzi P. (2022). Environmental Assessment of Rubber Recycling through an Innovative Thermo-Mechanical Devulcanization Process Using a Co-Rotating Twin-Screw Extruder. J. Clean. Prod..

[79] Stockwell C.E., Yokelson R.J., Kreidenweis S.M., Robinson A.L., Demott P.J., Sullivan R.C., Reardon J., Ryan K.C., Griffith D.W.T., Stevens L. (2014). Trace Gas Emissions from Combustion of Peat, Crop Residue, Domestic Biofuels, Grasses, and Other Fuels: Configuration and Fourier Transform Infrared (FTIR) Component of the Fourth Fire Lab at Missoula Experiment (FLAME-4). Atmos. Chem. Phys..

[80] Karabork F., Pehlivan E., Akdemir A. (2014). Characterization of Styrene Butadiene Rubber and Microwave Devulcanized Ground Tire Rubber Composites. J. Polym. Eng..

